# Core Outcome Sets (COS) related to pregnancy and childbirth: a systematic review

**DOI:** 10.1186/s12884-021-04164-y

**Published:** 2021-10-09

**Authors:** Marie Österberg, Christel Hellberg, Ann Kristine Jonsson, Sara Fundell, Frida Trönnberg, Alkistis Skalkidou, Maria Jonsson

**Affiliations:** 1 Swedish Agency for Health Technology Assessment and Assessment of Social Services (SBU), Stockholm, Sweden; 2 Patient Representative, Linköping, Sweden; 3 Department of Women’s and Children’s Health, Uppsala University, Uppsala, Sweden

**Keywords:** Core outcome set, Consensus methods, Maternal health, Obstetric care, Pregnancy, Prenatal

## Abstract

**Background:**

Systematic reviews often conclude low confidence in the results due to heterogeneity in the reported outcomes. A Core Outcome Set (COS) is an agreed standardised collection of outcomes for a specific area of health. The outcomes included in a COS are to be measured and summarized in clinical trials as well as systematic reviews to counteract this heterogeneity.

**Aim:**

The aim is to identify, compile and assess final and ongoing studies that are prioritizing outcomes in the area of pregnancy and childbirth.

**Methods:**

All studies which prioritized outcomes related to pregnancy and childbirth using consensus method, including Delphi surveys or consensus meetings were included. Searches were conducted in Ovid MEDLINE, EMBASE, PsycINFO, Academic Search Elite, CINAHL, SocINDEX and COMET databases up to June 2021.

For all studies fulfilling the inclusion criteria, information regarding outcomes as well as population, method, and setting was extracted. In addition, reporting in the finalized studies was assessed using a modified version of the Core Outcome Set–STAndards for Reporting.

**Results:**

In total, 27 finalized studies and 42 ongoing studies were assessed as relevant and were included. In the finalized studies, the number of outcomes included in the COS ranged from 6 to 51 with a median of 13 outcomes. The majority of the identified COS, both finalized as well as ongoing, were relating to physical complications during pregnancy.

**Conclusion:**

There is a growing number of Core Outcome Set studies related to pregnancy and childbirth. Although several of the finalized studies follow the proposed reporting, there are still some items that are not always clearly reported. Additionally, several of the identified COS contained a large number (*n* > 20) outcomes, something that possibly could hinder implementation. Therefore, there is a need to consider the number of outcomes which may be included in a COS to render it optimal for future research.

**Supplementary Information:**

The online version contains supplementary material available at 10.1186/s12884-021-04164-y.

## Background

Well- designed and conducted clinical trials, mainly randomised controlled trials (RCTs), are used to establish the effectiveness of different interventions through comparison of outcomes. However, when research results are later synthesised in systematic reviews, it becomes clear that studies often overlook outcomes of importance to patients, that different outcomes are assessed and that different methods or timepoints for assessment are used. This has a negative impact on the certainty of the findings in systematic reviews, thus contributing to research waste. As a result, the scientific evidence to support many treatment procedures is attenuated [1, 2].

To overcome these problems, the core selection of outcomes and measurement properties in studies need to be standardised. Described and promoted by the Core Outcome Measures in Effectiveness Trials (COMET) initiative group in 2010, Core Outcome Sets (COS) have increasingly been developed for various conditions over time. According to COMET “A COS is a minimum set of outcomes to be selected, measured, and reported in trials of a specific condition” [3]. These are typically developed by identifying and describing the outcomes used in current research (primary studies as well as systematic reviews) and then allowing stakeholders to prioritize among these outcomes by using a consensus process. When a COS has been agreed on, the purpose is that researchers use it in all studies within that condition, adding further outcomes if they wish.

The aim of developing and implementing COS is that the results of various studies will be more readily comparable and collated, reinforcing the basis of decisions, to benefit patients and healthcare personnel.

In the research fields of women’s health and neonatal health, an international network, called CoRe Outcomes in Women’s and Newborn health (CROWN), has been established [4]. It is led by journal editors, and aims to address the widespread, unwarranted variation in reporting of outcomes, which makes comparison between and combination of results across studies difficult, if not impossible. This initiative might explain the rather large production of COS in this area. This was also illustrated in a previous systematic review with focus on COS related to the health of women and new-born published in 2017 [5]. This review identified four finalized COS and an additional 49 ongoing COS, thus motivating an updated systematic review to investigate any new activity on the topic.

The aim of this article is to systematically identify and describe ongoing and finalized COS projects (including all projects where outcomes where prioritized), within the field of pregnancy and childbirth.

## Methods

The study consisted of a systematic literature review undertaken to analyse and summarize ongoing and finalized COS projects (including all projects where outcomes where prioritized), within the area of pregnancy and childbirth. The literature search was conducted in June 2019, and an updated search was conducted in June 2021.

### Protocol and registration

This manuscript is an updated version of a governmental report published by SBU 2020 [6].

A project plan was established a priori and registered at SBU, the PROSPERO database (CRD420201490792020) [7] as well as the COMET database [8]. This systematic review was conducted and reported in accordance with the PRISMA statement [9].

### Eligibility criteria

The criteria for eligibility were outlined according to the PICOS model (Population, Intervention, Comparator, Outcome and Study design) and included the following characteristics:

#### Population

Pregnant women, women during labour and birth, women who suffer from an injury or other complications related to childbirth, women or men suffering from a mental health disorder during pregnancy or during or after childbirth.

#### Intervention

No restriction.

#### Control

Not applicable.

#### Outcome

A list of outcomes included in the COS.

#### Study design

Ongoing or finalized original studies where outcomes were prioritized using some form of consensus. No restriction applied to publication status.

#### Language

English and Scandinavian languages.

#### Exclusion criteria

- Systematic reviews of outcomes
- Qualitative studies identifying important outcomes, without any form of prioritization
- COS studies focusing only on the child (no outcomes related to the women)
- COS studies relating to interventions/conditions prior to pregnancy, such as in vitro fertilization, contraceptives use etc.

### Information sources and search strategy

Studies were identified by searching electronic databases and by scanning the reference lists of studies meeting the eligibility criteria. The electronic databases MEDLINE, Embase, PsycINFO, Academic Search Elite, CINAHL with Full Text and SocINDEX with Full Text and the Core Outcome Measures in Effectiveness Trials (COMET) Initiative database were searched up to June 2021. Electronic searches were conducted using a combination of medical subject headings (MeSH) and relevant text word terms related to the population, in combination with different terms related to Core Outcome Set. (For detailed information about the search strategies, Additional file 1) In addition, the CROWN website was hand searched [4].

### Identification of studies

Two reviewers (MÖ and CH) independently screened the titles and abstracts for eligibility. The abstracts were screened and rated using the scanning tool Rayyan, available online [10]. Full text articles were retrieved and reviewed to determine eligibility, independently and in duplicate by two authors (CH and MÖ). Disagreements were resolved by discussion. The reference lists of studies meeting the eligibility criteria were screened for additional relevant studies.

### Description of methodology in included studies

In order to check the description of the methodology in the included studies, a checklist was compiled using the items from the COS-STAR reporting guide (Additional file 2). The COS-STAR is developed as a reporting checklist and is not developed or validated as a quality assessment tool [11]. However, no such tool exists, and the project management team decided to use this existing reporting guide to investigate the COS. The involvement of relevant stakeholders is an important feature of COS development; therefore, one further question was added to the checklist: “Are researchers as well as healthcare providers and patients included in the development process?” (Additional file 2). Two of the authors (CH and MÖ) independently reviewed the included articles according to the checklist. Disagreements were resolved by discussion.

### Data items

The following information was extracted from the included trials: Population, intervention, setting for intended use, consensus method, number and characteristics of participants, number of outcomes at the start of the project and number of outcomes in the final COS, consensus criteria and the degree of compliance with COS-STAR.

Data were extracted from each included study and tabled by one reviewer. A second reviewer audited the data extraction. Any disagreements were resolved by discussion.

Since the results were not suitable for synthesis, the included studies are described narratively.

### Patient involvement

A patient representative with lived experience of birth trauma, birth injury and postpartum depression (FT) was included in the project management group to ensure patient input into all aspects of the work.

## Results

### Eligible studies

**Fig. 1:**
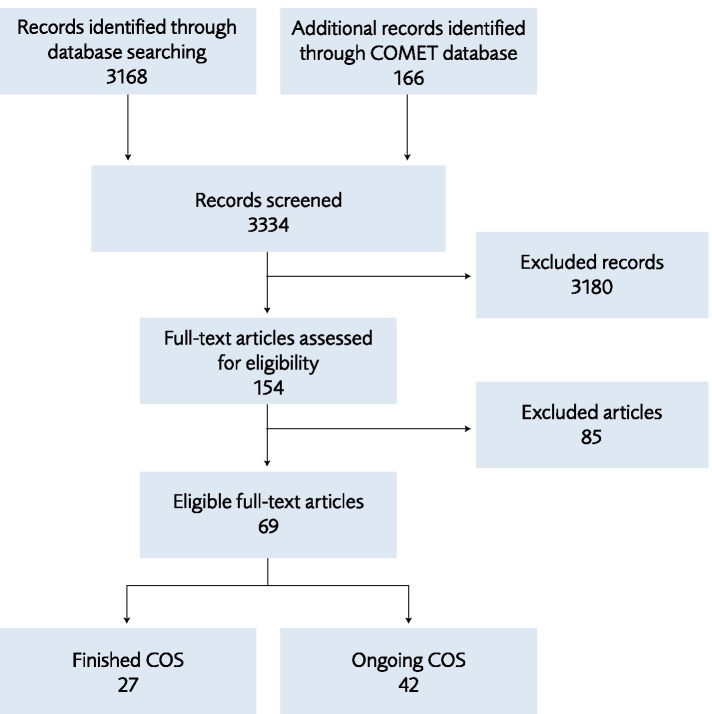
Study flow diagram

The literature search yielded a total of 3334 citations: after review of the abstracts, 154 were assessed in full. Eighty-five studies which did not meet the inclusion criteria were excluded, leaving 69 relevant studies. Of these, 27 [12–38] were finalized studies with prioritized outcomes and 42 were COS protocols, where the final COS was not yet published (Fig. 1) [39–80].

Information about the included finalized COS studies is presented in Table 1 and the outcomes included in the final COS are presented in Table S3. Excluded studies and the reason for exclusion can be found in Table S1. Forty of the 42 ongoing COS studies were identified through the COMET database and a published full protocol was identified for 14. The ongoing studies are described in Table S2.

**Table 1:** Characteristics of included studies

**Author, Year Ref**	**Subject**	**Study objective**	**N outcomes in COS**	**Methods**	**Consensus criteria for inclusion in the COS**	**Stakeholders included**	**Participants: N included, % answering all surveys, N at meeting**	**Protocol (Y/N)** **Comet Registration number**	**Compliance with COS-STAR**
**Pregnancy**									
Bashir et al. 2021 [12]	Gestational diabetes for treatment trials	A Core Outcome Set	11^a^	Five round Delphi survey and consensus meetings	Agreement by three-quarters of those present	Researchers and clinicians from Qatar, North America, Europe, and Australia^b^	Not stated	N 1790	Some details not reported
Meissner et al. 2021 [31]	Pregnancy registries in rheumatology	A Core data set for registries	51^a^	A two-round Delphi survey and consensus meeting	> 70% scored as critically important in Delphi and > 50% of votes in meeting	Multidisciplinary stakeholders from 14 different countries^b^	64, 88%, 12	N 1710	Some details not reported
Duffy et al. 2020 [19]	Pre-eclampsia research	A Core Outcome Set	22^a^	A Three-round Delphi survey and consensus meeting^c^	70%/ 15%^d^ in each stakeholder group	Patients, clinicians, researchers from 56 nations	432, 63%,11	Y^e^ 588	Good
Egan et al. 2020 [20]	Gestational diabetes mellitus prevention and treatment	A Core Outcome Set	14	A three-round Delphi survey and consensus meeting	70%/ 15%^d^ in each stakeholder group and ≥ 70% voted ‘yes in meeting	Patient representatives, researchers and clinicians from 27 countries	173^f^, 59%, 23	Y^e^ 686	Good
Jansen et al. 2020 [27]	Hyperemesis gravidarum research	A Core Outcome Set	24	A two-round Delphi survey and consensus meeting^c^	70%/ 15%^d^ in each stakeholder group	Patient representatives, researchers and clinicians from 22 countries	277, 45%,20	N 805	Good
Bogdanet et al. 2019 [14]	Follow-up at 1 year and beyond of women with gestational diabetes	A Core Outcome Set	9	A three-round Delphi survey and consensus meeting	70%/ 15%^d^	Patients, clinicians, researchers, policy makers and others from 33 nations	835, 20%, 20	Y^e^ 1031	Good
Bunch et al. 2018 ( [16]	Monitor the quality of maternity care	Developing a set of consensus indicators	14	A two-round Delphi survey and consensus meeting	70%/ 15%^d^	Service designers, providers and users from England^b^	101, 71%, 19	N	Some details not reported^g^
Nijagal et al. 2018 [32]	Pregnancy and childbirth	Standardized outcome measures	23	A series of nine teleconferences, incorporating a modified Delphi process	Outcome domains thought to be “critical” (7–9) by at least 70%	Consumer representatives and international experts, researchers and patient advocacy from 8 nations	21, 73%, NA	N 875	Some details not reported
Egan et al. 2017 [21]	Pre-pregnancy care for women with pregestational diabetes	A Core Outcome Set	17	A three-round Delphi survey and consensus meeting	70%/ 15%^d^	Clinicians’ patient’s policy makers, researchers, advocates on behalf of those with diabetes and others from 24 nations	151^f^, 67%, 14	Y^e^ 692	Good
Al Wattar et al. 2016 [11]	Epilepsy in pregnancy	A Core Outcome Set	31	A three-round Delphi survey and consultation meeting^i^	70%/ 15%^d^ (used a 5-point scale)	Healthcare professionals, and patient representatives from UK.^b^	99^f^, 49%, 15	Y^h^ 393	Good
Rogozinska et al. 2016 [34]	The effects of diet and lifestyle in pregnancy	Composite outcomes for individual patient data (IPD) meta-analysis	Maternal: 6 Neonatal: 4	A two-round Delphi survey	Considered to be critically important by the (score > 7) by the panel	Researchers from the International Weight Management in Pregnancy collaborative network from 11 nations^b^	26, 96%, NA	N	Significant details not reported
van ʼt Hooft et al. 2016 [37]	Interventions to Prevent Preterm Birth	A Core Outcome Set	13	A two-round Delphi survey and consensus meeting	70%/ 15%^d^ in each stakeholder group	Parents, clinicians, and researcher from 25 nations	228, 76%, 29	Y^h^ 603	Good
Fong et al. 2014 [23]	Management of late-onset pre-eclampsia	Maternal and neonatal composite outcomes	Maternal: 7 Neonatal: 3	A two-round Delphi survey	A median score of ≤4 and indicated consensus (IQR ≤2) for evaluation in the third stage. (5-point scale)	Practising senior clinicians and clinical academics from the United Kingdom^b^	44, 90% maternal outcomes 75% neonatal outcomes, NA	N 716	Significant details not reported
Saldanha et al. 2013 [35]	Gestational diabetes mellitus	Identify research needs, including which outcomes to measure, from a systematic review	Maternal: 17 Neonatal:13	Delphi method	Not specified	Clinicians, primary researchers, research funders, insurers, and patients or patient representatives from 1 nation	9	N	Significant details not reported
Bennett et al. 2012 [13]	Gestational diabetes mellitus	Identify research needs, including which outcomes to measure	Medication: 8 Delivery:8	One- survey	Appearing in the top 3 list of two or more of the nine stakeholders	Clinical experts^b^	20, NA, NA	N 527	Significant details not reported
Mehra et al. 2012 [30]	Weight management interventions in pregnancy	Prioritisation of outcomes	Presents the top 5	A two-round Delphi survey	Not enough information provided	20 Consultants from 2 nations^b^	20 participants	N	Significant details not reported
Devane et al. 2007 [17]	Evaluating maternity care	A Core Outcome Set	48	A three-round Delphi survey	Both a mean above the overall group mean for all outcomes combined and rated 4–5 by at least 70%	Healthcare professionals and patients from 28 nations.	320, 48%, NA	N 108	Some details not reported^g^
**Misscarriage, abortion and stillbirth**									
Kim et al. 2021 828)	Interventions to prevent stillbirth	A Core Outcome Set	11	A two-round Delphi survey and consensus meeting^c^	70%/ 15%^d^	Clinicians, researchers and parents	129^f^ 69%, 20	Y^h^ 982	Some details not reported
Fiala et al. 2018 [22]	First trimester medical termination of pregnancy	Definitions of outcomes	NA	Consensus meeting	Not specified	Clinicians, researchers and members of the pharmaceutical industry from Europe^b^	Number of participants not clearly stated	N	Significant details not reported^g^
**Mental Health**									
Hellberg et al. 2021 [26]	Treatment studies of perinatal depression	A Core Outcome Set	9	A three-round Delphi survey and consensus meeting^c^	70%/ 15%^d^ in each stakeholder group	Clinicians, researchers and patients from 13 countries	222, 55%,13	Y 1421	Good^j^
**Labour and delivery and complications**									
Gachon et al. 2021 [24]	Mediolateral episiotomy on obstetric anal sphincter injury during operative vaginal delivery	A Core Outcome Set for a one-year observational French study	51 outcomes 63 variables	A three-round Delphi survey^i^	at least 50% of respondents considered the item as essential	Obstetricians and women in the community	Clinicians: 109, 37%, NA Women: 24, 63%, NA	N	Some details not reported^g^
Briscoe et al. 2019 [15]	Cesarean deliveries with infectious morbidity outcomes	A Core Outcome Set	6	A two-round Delphi survey	Majority of respondents	Systematic review authors^b^	41, 34%, NA	N 763	Significant details not reported
Dos Santos et al. 2018 [18]	Induction of labour	A Core Outcome Set	28	A two-round Delphi survey and consensus meeting	70%/ 15%^d^	Midwives, obstetricians, neonatologists, and women’s representatives	159, 45%, 20	Y^h^ 695	Good
Meher et al. 2019 [29]	Prevention and treatment of postpartum haemorrhage	Two Core Outcome Sets	Prevention: 9 Treatment: 12	A two-round Delphi survey and consensus meeting	70%/ 15%^d^ in each stakeholder group	Healthcare professionals and women’s representatives from 36 nations	Prevention: 205, 74%, 36 Treatment: 197, 73%, 36	Y^h^ 706	Good
**Fetal / neonatal**									
Healy et al. 2019 [25]	Prevention and treatment of fetal growth restriction	A Core Outcome Set	22	A three-round Delphi survey and consensus meeting	70%/ 15%^d^	Healthcare providers, researchers/academics, members of the public from 36 nations	238, 45%, not specified	Y^e^ 689	Good
Perry et al. 2019 [33]	Treatments for twin-twin transfusion syndrome.	A Core Outcome Set	12	A three-round Delphi survey and consensus meeting^c^	70%/ 15%^d^	Healthcare professionals, researchers and patients or relatives of patients from 29 nations	103^f^, 85%, 16	Y^e^ 1392	Good
Townsend et al. 2019 [36]	Management of selective fetal growth restriction in twins.	A Core Outcome Set	11	A three-round Delphi survey and consensus meeting^c^	A median score of 8 after the third round were taken forwards for discussion	Clinicians, obstetricians, fetal medicine specialists, neonatologists, and midwives, researchers, and parents or patients from 23 nations.	102^f^, 86%, 19	Y^e^ 998	Good

^a^Also includes how to measure the selected outcomes in the same paper or in an additional publication

^b^No or limited patient involvement

^c^Using the modified nominal group technique

^d^An outcome was included in the COS if at least 70% of participants scored the outcome as critically important (usually 7–9) and < 15% to score the outcome as not important (usually 1–3)

^e^Protocol published as a scientific paper

^f^Does not specify the number of non-responders from the first survey

^g^No systematic review of outcomes done prior to outcome prioritization/COS development

^h^Mention protocol in paper but no reference is given

^i^Uses separate surveys for patients

^j^Study done by the authors of current publication

### Published core outcome sets

In total, 19 of the finalized studies had COS development as the main purpose [12, 15, 16, 18, 19, 22, 26, 30, 31, 34, 37, 38] (Table 1). Of the eight remaining included studies, the main aim of the studies varied somewhat, but they all included prioritization of outcomes [14, 17, 23, 24, 32, 33, 35, 36]. Two articles had as primary aim to prioritize future research questions, including prioritizing the outcomes to be measured [14, 36]. Two articles examined which outcomes to include in a composite outcome and other studies considered outcomes to be included and assessed in clinical follow-up of patients or reporting to registers [17, 23, 24, 32, 33, 35]. Six of the 27 studies were not registered in the COMET databases (Table 1). Of those registered only two did not yet provide a link to the published paper [27, 37].

**Fig. 2:**
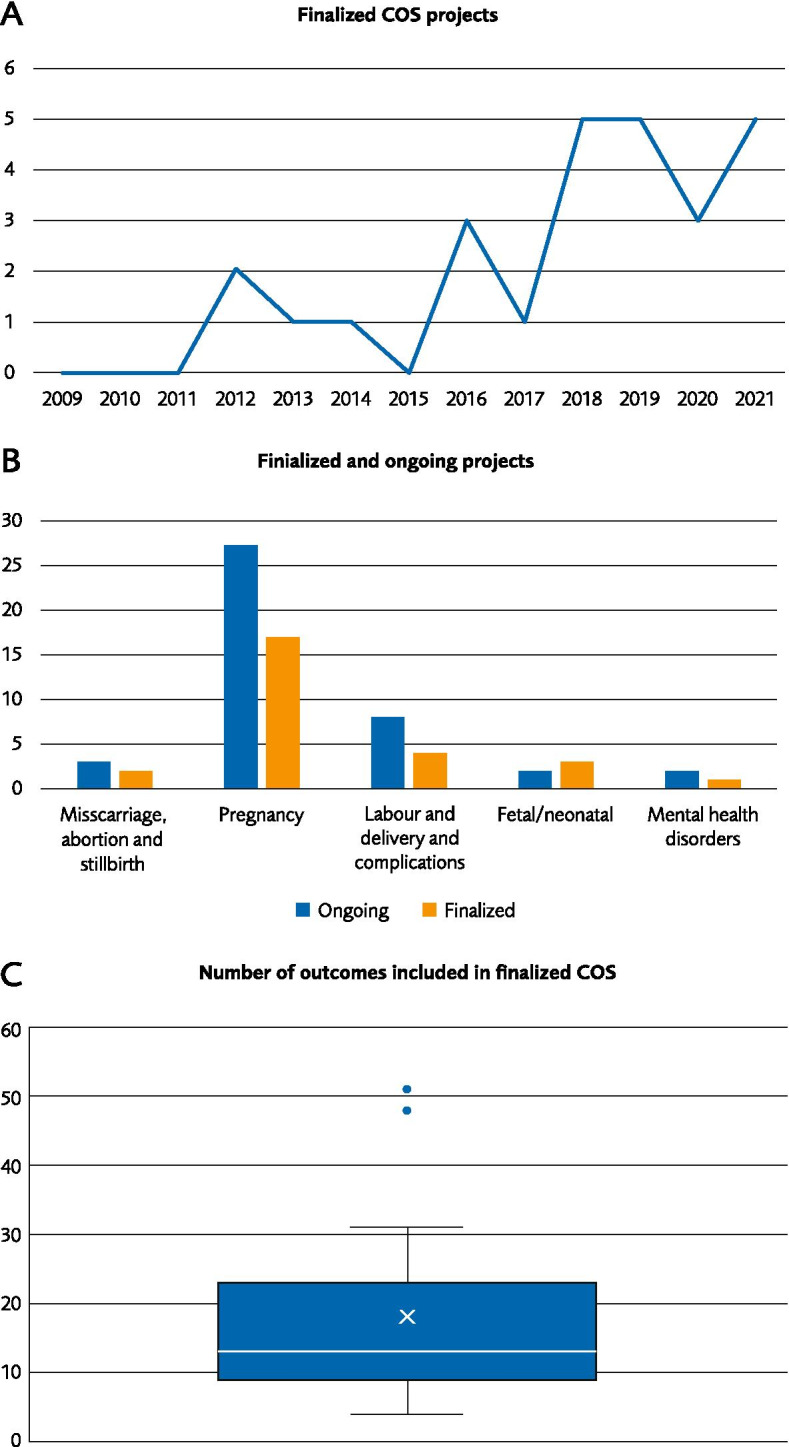
Description of included final and ongoing COS studies. **A** Number of final COS studies by year of publication. **B** Number of final and ongoing COS studies categorized by sub-topics and **C** Boxplot depiction of number of outcomes in the final COS (median 13 and mean 18). Data from studies with the aim of prioritizing which outcomes to include in a composite outcome or only presenting the top outcomes in the COS are not included in the boxplot

Of the finalized studies, all were published after 2007 and 67% were published during 2018–2021 (Fig. 2A). The large number of ongoing COS projects identified indicates a high degree of activity in the field.

Categorisation of studies (Fig. 2B), disclosed that most COS, both finalized and ongoing, focus on pregnancy and pregnancy-related complications and conditions. There are few COS studies focusing on labour, birth and physical conditions associated with giving birth. Also, a limited number of studies, one finalized and two ongoing, were identified relating to mental health during pregnancy or after childbirth [27, 39, 49]. Since the focus of our review is Core Outcome Sets relating to pregnancy and childbirth and not neonatal and fetal aspects only a few finalized COS have been included which presents both outcomes related to the women as well as the featus/newborn. Therefore, the number of finalized COS relating to fetal/neonatal should be interpreted with caution. And we are aware of at least two additional finalized COS which were excluded from this review since the focus is on the new-born [81, 82].

### Use of method and representation

Most of the finalized studies described a 2 or 3 round Delphi survey, followed by a face-to-face consensus meeting to finalize the COS (Table 1). However, some finalized studies included only Delphi surveys and one study by Fiala et al. only undertook a consensus-meeting (Table 1) [23].

The consensus criteria most commonly used for an outcome to be included in the COS was the “70/15 rule” (more than 70% rates the outcome as critically important and less than 15% rates it as not important) (Table 1). The number of outcomes included in the COS ranged between 6 and 51 with a median number of 13 (Fig. 2C, Table 1). Only a few studies had less than 10 outcomes in the final COS. Only one study mentioned that a possible maximum limit to the number of outcomes to be included in the COS had been determined or discussed in advance, in order to enable implementation and feasibility in research [27]. Six studies described using a “modified nominal group technique” during the consensus meeting in order to reduce the number of outcomes (Table 1) [20, 27–29, 34, 37].

Researchers were included in all identified studies and healthcare personnel in the majority of them. Patients were sometimes not included at all in the process or only partly included [12–14, 16, 17, 23, 24, 31, 32, 35]. Some examples of limited patient inclusion: Al Wattar et al. [12] who used a separate survey consisting of only one round for patients; Bunch et al. [17] where patients were included in the Delphi survey, but not in the consensus meeting and Bennet et al. [14] where two persons served as proxies for patients. Most of the finalized studies involved international participation (Table 1).

Thirteen of the studies were assessed as complying well with the COS-STAR criteria in most categories [12, 15, 19–22, 26–28, 30, 34, 37, 38], seven showed some deviations [13, 17, 18, 25, 29, 32, 33] and seven of the studies were assessed as having major shortcomings in reporting [14, 16, 23, 24, 31, 35, 36]; however, five of those were published prior to the publication of COS-STAR (Table 1 and Table S4). Most of the finalized studies lacked information about whether outcomes had been excluded at some stage or if outcomes had been merged. Only one study mentioned whether they deviated from the study protocol in any way [27].

## Discussion

### Main findings

Although there are examples of well-established COS such as Outcome Measures in Rheumatology (OMERACT) for rheumatoid arthritis, they are still relatively rare in most medical fields [83]. This review of pregnancy and childbirth revealed that most of the COS are developed for physical conditions that occur during pregnancy. A minority of the ongoing or existing COS focus on mental health. There are also a few COS on intrapartum care, for such conditions as slow progress in labour, trial of labour after previous caesarean section and postpartum endometritis. One of the topics for which most COS have been compiled is the field of physical conditions and complications during pregnancy.

It is important to consider how many outcomes a COS can include and still be applicable and useful for research. This systematic review discloses that the COS identified range between 6 and 51 outcomes with a median of 13 outcomes. Only a few of the included finailzed COS had less than ten outcomes. None of the identified studies discussed the relationship between the number of outcomes in the COS and the median number of outcomes in the studies for which the COS is intended for. Nor did any of the protocols suggest a possible maximum limit to the number of outcomes that might be included in the intended COS. In order to increase the implementation of the developed COS, it is important to consider how the number of outcomes included will affect the usefulness of the COS. A limitation of the number of outcomes might increase the likelihood that the COS are indeed applied in future research. There are consensus processes that include several stakeholders, where a pre-set goal is communicated from start to the participants. The prioritization of research questions by James Lind Alliance is one example of such a process, where a top-ten list of research questions are to be agreed upon [84]. Surely, such a limit might impact the COS development process and it might be even more important to balance the influence between different stakeholders along the process, especially in consensus meetings. Nevertheless, a limit might also be a positive contributing factor in the process, putting pressure on the participants to limit their choices of the most important outcomes.

Another aspect that might need further discussions and guidance is how extensive the scope of a COS can be. As illustrated in this systematic review, some COS are more generic, covering broad areas, as for example the whole maternal care period, while others are more precise and niched, as for example twin to twin transfusion syndrome. This might result in numerous overlapping COS, and potentially introduces challenges when researchers are faced with more than one COS to comply with.

It is also important to note that the development of a COS which focuses on *what* to measure may need to be followed by decisions about *how* and *when* to measure these outcomes. Even if the outcomes themselves are consistent across the studies, lack of consistency in how or when outcomes have been measured can undermine efforts by systematic reviewers to compare, contrast and combine the results of multiple studies. Unfortunately, only a few of the identified COS mentioned how and when to measure the outcomes in the developed COS.

### Strengths and limitations

Some limitations to the systematic review should be noted. In the systematic review, we checked compliance to COS-STAR in the included studies (Additional file 2) [11]. Another possibility would have been to check how well the different projects adhered to the COS-STAD guidelines [85]. However, none of these guidelines was developed to check methodological quality. For instance, both recommendations discuss that one should describe/report a scoring process and consensus definition a priori, but not if the process/definition was suitable. In addition, the COS-STAD does not include items concerning the availability of a protocol, if any adjustments were made to it, or if conflicts of interest and ethical approval existed [85]. This guided our decision to check for how well the published studies reported their findings in accordance with COS-STAR. In addition, we also checked if all relevant stakeholder groups were included in the development. However, it would have been optimal to be able to assess the methodological quality of the included studies using a tool developed for this purpose. We believe that the development of such a tool is desirable and that some of the questions used in this article (Additional file 2) could be helpful. Further, in this systematic review, we decided to have an inclusive approach and might have included studies that were not principally intended for research use, but for other purposes, such as clinical follow-up.

A strength of this study is that it is methodologically sound and robust, and all results have continuously been reviewed by experts from the Swedish Agency for Health Technology Assessment and Assessment of Social Services (SBU), as well as by external reviewers. Another strength is the attempt to assess the reporting of the included COS using an assessment tool based on the COS-STAR reporting guide (Additional file 2).

### Interpretation

In 2017, Duffy et al. published a systematic review of published and ongoing COS related to the health of women and newborns [5]. The scope of their paper is somewhat broader, including conditions other than those related to pregnancy and childbirth. In all, they identified four finalized COS, of which three were related to pregnancy and childbirth. In the last years, a substantial number of COS have been finalized and 42 ongoing studies have been identified.

## Conclusion

This systematic review discloses an increasing number of COS for pregnancy and childbirth. This is gratifying and is hopefully leading to studies which focus on important outcomes and research that are more readily synthesised in systematic reviews, thus increasing evidence in support of interventions. The review reveals that a large number of the ongoing and finalized COS studies address physical conditions and complications during pregnancy. There was a lack of COS for birth-related studies. Only a few COS were identified for perinatal mental health.

## Supplementary Information

**Additional file 1.** Search strategies.

**Additional file 2.** Checklist for included studies.

**Additional file 3: Table S3.** Included studies.

**Additional file 4: Table S1.** Excluded studies.

**Additional file 5: Table S2.** Ongoing COS studies.

**Additional file 6: Table S4.** Compliance with COS-STAR Items.

## Data Availability

The datasets used and/or analysed during the current study are available from the corresponding author on reasonable request.

## Abbreviations

COMET: Core Outcome Measures in Effectiveness Trials Initiative
COS: Core Outcome Set/ Sets
COS-STAD: The Core Outcome Set-STAndards for Development
COS-STAR: Core Outcome Set–STAndards for Reporting
CROWN: Core Outcomes in Women’s and Newborn Health
RCT: Randomised Controlled Trial
SBU: Swedish agency for health technology assessment and assessment of social services
HTA: Health Technology Assessment
