# Supplementary material for: Core Outcome Sets (COS) related to pregnancy and childbirth: a systematic review
Source: BMC Pregnancy Childbirth. 2021 Oct 9;21:691. doi: 10.1186/s12884-021-04164-y (PMC8501579; doi:10.1186/s12884-021-04164-y)
Supplement: Supplementary file 1 — Additional file 1. Search strategies. [file 12884_2021_4164_MOESM1_ESM.docx]

**Additional file 1. Search strategies**

**Embase via Elsevier 26 June 2019**

**Title: Core outcomes sets (COS) in obstetric care**

| Search terms | | Items found |
| --- | --- | --- |
| **Intervention:** | | |
| 1. | 'core outcome set'/exp | 59 |
| 2. | "core outcome*":ti,ab or "core event*":ti,ab | 1620 |
| 3. | ((core or composite or harmonise* or harmonize* or minimal or prioriti* or standard or standards or standardise* or standardize* or set or sets) NEAR/2 outcome*):ti,ab | 17358 |
| 4. | 1-3 (or) | 17425 |
| **Population:** | | |
| 5. | 'childbirth'/exp or 'obstetric anesthesia'/exp or 'obstetric procedure'/exp or 'obstetrics'/exp or 'pregnant woman'/exp or 'pregnancy'/exp | 1056620 |
| 6. | abortion:ti OR antenatal:ti OR antepartum:ti OR birth:ti OR caesarean:ti OR childbirth:ti OR delivery:ti OR fertilization:ti OR gestation:ti OR labor:ti OR labour:ti OR maternal:ti OR neonatal:ti OR obstretric*:ti OR perinatal:ti OR peripart*:ti OR postnatal:ti OR postpartum:ti OR pregnan*:ti OR puerperium:ti OR preterm:ti | 810053 |
| 7. | 5 OR 6 | 1398844 |
| **Combined sets** | | |
| 8. | 4 AND 7 AND ([medline]/lim OR [pubmed-not-medline]/lim) | 1918 |
| 9. | 4 AND 7 AND [embase]/lim AND ([danish]/lim OR [english]/lim OR [norwegian]/lim OR [swedish]/lim) | 979 |
| **10.** | 9 NOT 8 | **935** |

The search result, usually found at the end of the documentation, forms the list of abstracts.

*OBS!
Sökdokumentationen räknas som arbetsmaterial och får inte spridas utanför projektgruppen innan rapporten publiceras. Om sökstrategin i sin helhet används i andra sammanhang (t.e.x vid publicerande av artikel) bör man hänvisa till den publicerade sökdokumentationen på* [*www.sbu.se*](http://www.sbu.se)

*OBS!
Sökdokumentationen räknas som arbetsmaterial och får inte spridas utanför projektgruppen innan rapporten publiceras. Om sökstrategin i sin helhet används i andra sammanhang (t.e.x vid publicerande av artikel) bör man hänvisa till den publicerade sökdokumentationen på* [*www.sbu.se*](http://www.sbu.se)

*OBS!*

*Sökdokumentationen räknas som arbetsmaterial och får inte spridas utanför projektgruppen innan rapporten publiceras. Om sökstrategin i sin helhet används i andra sammanhang (t.e.x vid publicerande av artikel) bör man hänvisa till den publicerade sökdokumentationen på* [*www.sbu.se*](http://www.sbu.se)

/de= Term from the EMTREE controlled vocabulary

/exp= Includes terms found below this term in the EMTREE hierarchy

/mj = Major Topic

:ab = Abstract

:au = Author

:ti = Article Title

:ti:ab = Title or abstract

* = Truncation

“ “ = Citation Marks; searches for an exact phrase

**Medline (Ovid) June 26 2019**

**Title: Core outcomes sets (COS) in obstetric care**

| Search terms | | Items found |
| --- | --- | --- |
| **Intervention:** | | |
|  | ("core outcome*" or "core event*" or ((core or composite or harmoni#e* or minimal or prioriti* or standard or standards or standardi#e* or set or sets) adj2 outcome*)).mp. | 10749 |
| **Population:** | | |
| 2. | exp Pregnancy Complications/ or exp Pregnancy/ or exp Obstetric Surgical Procedures/ or exp Anesthesia, Obstetrical/ or exp Analgesia, Obstetrical/ or exp Obstetrics/ or exp Pregnant Women/ or (abortion or antenatal or antepartum or birth or caesarean or caesarean or caesarean or childbirth or delivery or fertilization or gestation or labor or labour or maternal or neonatal or obstretric* or perinatal or peripart* or postnatal or postpartum or pregnan* or puerperium or preterm).ti | 1199069 |
| **Combined sets/Limits:** | | |
|  | 1 and 2 Limiters - Language: Danish, English, Norwegian, Swedish | **1028** |

The search result, usually found at the end of the documentation, forms the list of abstracts.

[MeSH] = Term from the Medline controlled vocabulary, including terms found below this term in the MeSH hierarchy

[MeSH:NoExp] = Does not include terms found below this term in the MeSH hierarchy

[MAJR] = MeSH Major Topic

[TIAB] = Title or abstract

[TI] = Title

[AU] = Author

[OT]= Other term

[TW] = Text Word

Systematic[SB] = Filter for retrieving systematic reviews

* = Truncation

**PsycINFO, Academic Search Elite, CINAHL with Full Text, SocINDEX with Full Text via EBSCO.
26 June 2019**

**Title: Core outcomes sets (COS) in obstetric care**

| Search terms | | Items found |
| --- | --- | --- |
| **Intervention:** | | |
|  | TI ( ("core outcome*" or "core event*" or ((core or composite or harmoni#e* or minimal or prioriti* or standard or standards or standardi#e* or set or sets) NEAR/2 outcome*)) ) OR AB ( ("core outcome*" or "core event*" or ((core or composite or harmoni#e* or minimal or prioriti* or standard or standards or standardi#e* or set or sets) NEAR/2 outcome*)) ) | 1414 |
| **Population:** | | |
| 2. | TI ( (abortion or antenatal or antepartum or birth or caesarean or caesarean or caesarean or childbirth or delivery or fertilization or gestation or labor or labour or maternal or neonatal or obstretric* or perinatal or peripart* or postnatal or postpartum or pregnan* or puerperium or preterm) ) OR AB ( (abortion or antenatal or antepartum or birth or caesarean or caesarean or caesarean or childbirth or delivery or fertilization or gestation or labor or labour or maternal or neonatal or obstretric* or perinatal or peripart* or postnatal or postpartum or pregnan* or puerperium or preterm) ) OR SU ( (abortion or antenatal or antepartum or birth or caesarean or caesarean or caesarean or childbirth or delivery or fertilization or gestation or labor or labour or maternal or neonatal or obstretric* or perinatal or peripart* or postnatal or postpartum or pregnan* or puerperium or preterm) ) | 2019640 |
| **Combined sets** | | |
|  | 1 and 2 | **110** |

The search result, usually found at the end of the documentation, forms the list of abstracts.

AB = Abstract

AU = Author

DE = Term from the thesaurus

MM = Major Concept

TI = Title

TX = All Text. Performs a keyword search of all the  database's searchable fields

ZC = Methodology Index

* = Truncation

“ “ = Citation Marks; searches for an exact phrase

**Comet initiative database:**

**Date of Search,"10/06/2019", updated ,"27/01/2020",**

Search Options,"search{Health Area - Disease Category{:Anaesthesia & pain control:Gynaecology:Pregnancy & childbirth:Urology:}}",
