# Supplementary material for: Core Outcome Sets (COS) related to pregnancy and childbirth: a systematic review
Source: BMC Pregnancy Childbirth. 2021 Oct 9;21:691. doi: 10.1186/s12884-021-04164-y (PMC8501579; doi:10.1186/s12884-021-04164-y)
Supplement: Supplementary file 2 — Additional file 2. Checklist for included studies. [file 12884_2021_4164_MOESM2_ESM.docx]

**Additional file 2. Checklist for included studies**

**Article**:_________________________________________________________

|  | Item | Question | Yes | No | Unclear |
| --- | --- | --- | --- | --- | --- |
| Title and abstract | 1a | Does the title contain information that the paper reports the development of a COS? |  |  |  |
|  | 1b | Does the abstract contain the key information, such as a list of the recommended outcomes in the COS? |  |  |  |
| Introduction | 2a | Is a background and explanation of the rationale for developing the COS provided? |  |  |  |
|  | 2b | Are a clear objective presented? |  |  |  |
|  | 3a | Is the *health condition(s)* and *population(s)* covered by the COS sufficiently described? |  |  |  |
|  | 3b | Is the *intervention(s)* covered by the COS sufficiently described? |  |  |  |
|  | 3c | Is the *setting(s)* in which the COS is to be applied sufficiently described? |  |  |  |
| Methods | 4 | Do the authors provide information about were the COS development protocol can be accessed |  |  |  |
|  | 5 | Is the rationale for which stakeholder groups that are involved and the eligibility criteria for participants described? |  |  |  |
|  | 6a | Are the information sources used to identify an initial list of outcomes provided? |  |  |  |
|  | 6b | Is it described, with reasons, how outcomes were dropped/combined if this was done? |  |  |  |
|  | 7 | Is a description for how the consensus process was undertaken provided? |  |  |  |
|  | 8 | Is a description for how outcomes were scored and summarised, provided? |  |  |  |
|  | 9a | Is a description of the consensus definition provided? |  |  |  |
|  | 9b | Is a description of the procedure for determining how outcomes were included or excluded from consideration during the consensus process provided |  |  |  |
|  | 10 | Is a statement regarding the ethics and consent issues for the study provided? |  |  |  |
| Results | 11 | Are any motivated deviations from the protocol and their eventual impact on the results described? |  |  |  |
|  | 12 | Are numbers and relevant characteristics of the people involved at all stages of COS development presented? |  |  |  |
|  | 13a | Are all outcomes considered at the start of the consensus process listed? |  |  |  |
|  | 13b | Are any new outcomes introduced or any outcomes dropped during the consensus process and the reasons for doing so described? |  |  |  |
|  | 14 | Is a list of the outcomes in the final core outcome set provided? |  |  |  |
| Discussion | 15 | Are limitations with the COS development process discussed? |  |  |  |
|  | 16 | Is an interpretation of the final COS in the context of other evidence, and implications for future research given? |  |  |  |
| Other information | 17 | Are sources of funding and role of funders given? |  |  |  |
|  | 18 | Are any conflicts of interest within the study team and how these were managed presented? |  |  |  |
| Representa-tion (Not included in COS-STAR) | 19 | Are researchers as well as health care providers and patients included in the development process? |  |  |  |
