# Supplementary material for: Core Outcome Sets (COS) related to pregnancy and childbirth: a systematic review
Source: BMC Pregnancy Childbirth. 2021 Oct 9;21:691. doi: 10.1186/s12884-021-04164-y (PMC8501579; doi:10.1186/s12884-021-04164-y)
Supplement: Supplementary file 3 — Additional file 3: Table S3. Included studies. [file 12884_2021_4164_MOESM3_ESM.docx]

Table S3 Included studies.

| First Author, Ref  Year | Population  Intervention  Setting | Number of outcomes:  -in first round  -added by -participants  -in final COS | Final COS | Compliance with COS-STAR  Comments |
| --- | --- | --- | --- | --- |
| Pregnancy | | | | |
| Bashir et al (1)  2021 | Women of age 16 years and above who were diagnosed  with GDM and were on GDM treatment after their diagnosis  Treatment  Clinical trials | 50  Not enough information provided  11 | Maternal metabolic outcomes:   - Average plasma glucose - Glycemic targets unmet - Adverse events related to treatment - Total weight gain in pregnancy (kg)   Fetal Outcomes:   - Birth weight (newborn's weight at birth) - Hypoglycemia within 1 h of birth - Neonatal composite morbidity and mortality   outcome  Pregnancy outcomes:   - Assisted labor/delivery (including cesarean) - Preterm delivery - Peripartum infection - PIH or preeclampsia/eclampsia | Some details form COS-STAR not reported  No protocol available.  No patients participated.  Number of participants in each step are not specified |
| Meissner et al (2)  2021 | Pregnancy and 28-neonatal phase in women with inflammatory rheumatic disease (IRD)  Pregnancy registries in reumathology | 143  14  51 | Maternal information:   - Demographics and risk behaviours (6 items) - IRD disease characteristics (11 items) - Prevalent comorbidities (1 item)   Pregnancy:   - Obstetrical history (7 items) - Course of current pregnancy (6 items) - Delivery/outcome of current pregnancy (6 items) - Neonatal outcomes (6 items)   Treatment:   - Treatment 12 months prior to conception (3 items) - IRD treatment during pregnancy and post partum (4 items) - Use of other treatments during pregnancy (1 item) | Some details form COS-STAR not reported  A very large number of outcomes included in the final COS  Only two patients participated in the process and unclear if they attended the consensus meeting.  No protocol available. |
| Duffy et al (3)  2020 | Pre-eclampsia  Clinical trials and systematic reviews | 86  Maternal: 14  Offspring: 8 | Maternal outcomes:   - Maternal mortality - Eclampsia - Stroke - Cortical blindness - Retinal detachment - Pulmonary oedema - Acute kidney injury - Liver capsule haematoma or rupture - Placental abruption - Postpartum haemorrhage - Raised liver enzymes - Low platelets - Admission to an intensive care unit required - Intubation and mechanical ventilation (not for childbirth)   Offspring outcomes:   - Stillbirth - Gestational age at delivery - Birth weight - Small-for-gestational-age - Neonatal mortality - Neonatal seizures - Admission to a neonatal unit required - Respiratory support | Good compliance with COS-STAR |
| Egan et al (4)  2020 | Gestational diabetes mellitus (GDM)  Interventions for prevention or treatment  Clinical trails | 190  (Prevention:74 outcomes  Treatment: 116 outcomes)  14 | Maternal outcomes:   - GDM diagnosis (relevant to GDM prevention - studies only) - Adherence to the intervention - Hypertensive disorders of pregnancy - Requirement and type of pharmacological therapy for hyperglycaemia - Gestational weight gain - Mode of birth   Neonatal outcomes:   - Birthweight - Large for gestational age - Small for gestational age - Gestational age at birth - Preterm birth - Neonatal hypoglycaemia - Neonatal death - Stillbirth | Good compliance with COS-STAR  Does not specify the number of non-responders from the first survey. |
| Jansen et al (5)  2020 | Hyperemesis gravidarum  Treatment  Clinical trails | 56  24 | Symptoms:   - Nausea - Vomiting - Inability to tolerate oral fluids or food - Dehydration - Weight difference   Laboratory findings:   - Electrolyte imbalance   Need for interventions to manage symptoms:   - Intravenous fluid treatment - Use of additional medication   Maternal service utilisation:   - Hospital treatment   Maternal quality of life and wellbeing:   - Treatment compliance - Patient satisfaction with treatment received - Maternal physical and/or mental and/or emotional wellbeing - Daily functioning   Maternal harm:   - Short term adverse effects of treatment - Long term adverse effects of treatment - Maternal death   Pregnancy complications:   - Pregnancy complications (miscarriage, thrombosis and bleeding, hypertensive disorders, gestational diabetes)   Termination of pregnancy:   - Termination of wanted pregnancy - Consideration of wanted pregnancy   Birth outcomes:   - Babies born preterm - Babies born small-for-gestational-age   Offspring outcomes:   - Congenital anomalies - Offspring death - Neonatal morbidity | Good compliance with COS-STAR  A very large number of outcomes included in the final COS |
| Bogdanet et al,(6)  2019 | Women with gestational diabetes treated with insulin and/or oral glucose-lowering agents  Follow-up at 1 year and beyond  Clinical trails | 121  10  9 | - Assessment of glycaemic status - Diagnosis of type 2 diabetes since the index pregnancy - Number of pregnancies since the index pregnancy - Number of pregnancies with a diagnosis of GDM since the index pregnancy - Diagnosis of prediabetes since the index pregnancy - BMI - Post-pregnancy weight retention - Resting blood pressure - Breastfeeding | Good compliance with COS-STAR |
| Bunch et al, (7)  2018 | Women in maternity care  Monitor the quality of maternity care | 125  19  14 | - Smoking rate at booking - Rate of birth without intervention - Caesarean section delivery rate in Robson group 1 women - Caesarean section delivery rate in Robson group 2 women - Caesarean section delivery rate in Robson group 5 women - Third-and fourth-degree tear rate among women delivering vaginally - Rate of postpartum haemorrhage of ≥1500 ml - Rate of successful vaginal birth after a single previous caesarean section - Smoking rate at delivery - Proportion of babies born at term with an Apgar score<7 at 5 minutes - Proportion of babies born at term admitted to the neonatal intensive care unit - Proportion of babies re admitted to hospital at<30 days of age - Breastfeeding initiation rate - Breastfeeding rate at 6–8 weeks | Some details form COS-STAR not reported  No reference to a protocol given  Patients are included in the panel but not represented at the final meeting |
| Nijagal et al, (8)  2018 | Women and infants  The care that they receive during pregnancy and the postpartum period | Not specified  Not specified  24 | - Maternal death - Still birth - Neonatal death - Maternal need for intensive care - Maternal length of stay - Late maternal complication - Transfusion - Spontaneous preterm birth - Iatrogenic preterm birth - Oxygen dependence - Neonate length of stay - Birth injury - Health related quality of life - Incontinence - Pain with intercourse - Success with breastfeeding - Confidence with breastfeeding - Mother-infant attachment - Confidence with role as a mother - Postpartum Depression - Satisfaction with the results of care - Confidence as an active participant in healthcare decisions - Confidence in healthcare providers - Birth Experience | Some details form COS-STAR not reported  No reference to a protocol given.  Only 2 consumers and 19 clinical expertise in the working group  A very large number of outcomes included in the final COS |
| Egan et al, (9)  2017 | Women with pregestational diabetes  Prepregnancy care  Clinical trials | 86  27  17 | - Healthcare professional review prior to conception - Smoking status at first antenatal visit - Use of folic acid preconception - Thyroid function at first antenatal visit - Use of potentially teratogenic medications at conception - Gestational age at first antenatal visit - BMI at first antenatal visit - BP at first antenatal visit - First trimester HbA1c - Perinatal mortality - Miscarriage - Congenital malformation - Preterm birth - Large for gestational age - Small for gestational age - Gestational weight gain - Severe maternal hypoglycaemia in first trimester | Good compliance with COS-STAR  Does not specify the number of non-responders from the first survey.  Only a few patient representatives (2/14) present in the workshop |
| Al Wattar et al, (10)  2016 | Epilepsy in pregnancy  Clinical trials | 70 grouped into 48  Not enough information provided  31 | Maternal   - Seizure control in pregnancy - Postpartum seizure control - Status epilepticus - Maternal mortality - Drowning - Sudden unexpected death in epilepsy - Postnatal depression - Maternal quality of life - Maternal anti-epileptic drug toxicity - Compliance with anti-epileptic drug intake   Offspring outcomes   - Major congenital abnormalities - Minor congenital abnormalities - Fetal anticonvulsant syndrome - Neurodevelopment - Autism spectrum disorder - Neonatal clinical complications - Admission to neonatal intensive care unit - Anthropometric measurements, including birthweight - Neonatal withdrawal symptoms - Neonatal haemorrhagic disease   Obstetric outcomes   - Live birth - Stillbirth - Miscarriage - Ectopic pregnancy - Termination of pregnancy - Maternal admission to high dependency or intensive care unit - Breastfeeding - Mode of delivery - Preterm birth - Pre-eclampsia   Eclampsia | Good compliance with COS-STAR  Patients participated in a separate survey which consisted of only one round  Only persons from UK represented  Does not specify the number of non-responders from the first survey.  A very large number of outcomes included in the final COS |
| Rogozinska et al, (11)  2016 | Pregnancy  Diet and lifestyle  Composite outcomes for individual patient data (IPD) meta-analysis | Maternal: 36  2  6 (condensed to 4)  Fetal and neonatal: 27  2  4 | The maternal composite outcome included   - Pre-eclampsia/pregnancy induced hypertension - gestational diabetes mellitus (GDM) - elective or emergency caesarean section - preterm delivery   The neonatal composite outcome included   - Intrauterine death - small for gestational age - large for gestational age   admission to a neonatal intensive care unit | Significant details from COS-STAR not reported  Aim is not a conventional COS development but a development of a composite outcome.  No reference to a protocol given  Only researchers included in the process |
| van ʼt Hooft et al, (12)  2016 | Pregnant women  Interventions to Prevent Preterm Birth  Clinical trials | 86 grouped into 29  2  13 | Related to pregnant women:   - Maternal mortality - Maternal infection or inflammation - Prelabor rupture of membranes - Harm to mother from intervention   Related to offspring:   - Gestational age at birth - Off-spring mortality - Birth weight - Early neurodevelopmental morbidity - Late neurodevelopmental morbidity - Gastrointestinal morbidity, infection - Respiratory morbidity - Harm to offspring from intervention | Good compliance with COS-STAR  Only a few patient (2/29) representatives present in the workshop |
| Fong et al, (13)  2014 | Late-onset preeclampsia  Management  Maternal and neonatal composite outcomes for trials | 21 maternal and 24 neonatal outcomes  8  Maternal composite outcome: 7  Neonatal composite outcome: 3 | The maternal composite outcome included   - maternal death - eclampsia - stroke or reversible ischaemic neurological deficit - pulmonary oedema - major obstetric haemorrhage - need for positive inotropic support - haemolysis - elevated liver enzymes and low platelets syndrome and placental abruption   The neonatal composite outcome included   - neonatal death - respiratory distress syndrome - needing ventilator support and neurological outcomes as cystic periventricular leukomalacia and grade III/IV intraventricular haemorrhage. | Significant details from COS-STAR not reported  Aim is not a conventional COS development but a development of a composite outcome.  No reference to a protocol given  No patients included in the process |
| Saldanha et al, (14)  2013 | Gestational diabetes mellitus  Antenatal drug treatment | Not enough information provided  Not enough information provided  Maternal:  17  Neonatal offspring:  13 | Oral agents compared with insulin:   - Chronic diseases (e.g., obesity and type 2 diabetes) in the offspring - Hypertensive disorders of pregnancy (e.g., GDM and pre-eclampsia) in the mother - Large for gestational age - Macrosomia in the neonate   Selective cesarean delivery or the choice of timing of induction:   - Cesarean delivery (primary cesarean and repeat cesarean) - Indication for cesarean delivery in the mother - Birth trauma (e.g., bone fractures and cerebral palsy) - Neonatal intensive care unit admission | Significant details from COS-STAR not reported  The aim is to article is to prioritize research need. For some of the research questions the outcomes to measure were also prioritized.  No reference to a protocol given |
| Bennett et al, (15)  2012 | Gestational Diabetes Mellitus  Medication and delivery management  Clinical trial | >20  NA  Medication management of GDM: 8  Delivery management for women with GDM: 8 | - Hypertensive disorders of pregnancy - Medication adherence - Large for gestational age and macrosomia - Gestational weight gain - Neonatal hypoglycemia - Neonatal intensive care unit admission - Chronic disease incidence in offspring - Postpartum incident type 2 diabetes mellitus or glucose intolerance/impaired fasting glucose mangaement - Cesarean delivery - Birth trauma - Neonatal intensive care unit admission - Patient-reported outcomes (e.g., patient preference, quality of life) - Complications of cesarean delivery (e.g., wound infection, wound dehiscence) - Vaginal delivery (spontaneous, operative) - Hypoxia/anoxia - Respiratory distress syndrome | Significant details from COS-STAR not reported  The aim is to article is to prioritize research need. For some of the research questions the outcomes to measure were also prioritized.  No patients included, but two members that served as proxy for the patient/consumer perspective |
| Mehra et al, (16)  2012 | Pregnancy weight management clinical trails  (Only available as a conference abstract) | Not enough information provided  Not enough information provided  Not enough information provided | Top 5 clinically important outcomes:   - Gestational diabetes - Preeclampsia - Gestational hypertension - Maternal admission to ITU/HDU - Venous thromboembolism | Not able to check compliance with COS-STAR  Conference abstract. Lot of information missing |
| Devane et al, (17)  2007 | Models of maternity care  Clinical trials | 263  73  48 | - Maternal death - Mode of birth - Neonatal death - Stillbirth - Type of labour onset - Neonatal admission to special care and/or intensive care unit - Birth injury to infant - Ruptured uterus - Postpartum haemorrhage - Mother requires admission to intensive care - Maternal postnatal readmission to hospital - Method of infant feeding - Vaginal birth after previous caesarean section - Gestational age at birth - Postnatal depression - Place of birth - Neonatal resuscitation required - Normal (i.e., physiological) birth without intervention - Oxytocin augmentation of labour - Anal sphincter damage - Hypoxic ischemic encephalopathy (a condition of injury to the brain) - Intrapartum hypertensive disorders of pregnancy - Hypertensive disorders of pregnancy - Puerperal psychosis - Maternal fecal incontinence - Neonatal readmission to hospital - Apgar score at 5 min - Trial of labour after previous caesarean delivery - Breastfeeding at 3 months - Maternal satisfaction (postnatal) - Infant birthweight - Neonatal fitting/seizures - Infant requiring intubation - Congenital anomaly (chromosomal, genetic, and/or structural) - Use of pharmacological analgesia/anesthesia - Maternal satisfaction (antenatal) - Postnatal hypertensive disorders of pregnancy - Maternal satisfaction (intrapartum) - Caesarean section wound infection - Pulmonary embolism - Intrauterine growth restriction - Preterm labour - Meconium aspiration - Intrapartum haemorrhage - Neonatal infection - Shoulder dystocia - HELLP = hemolysis, elevated liver enzymes, and low platelets - Birth asphyxia - Breastfeeding at discharge | Some details form COS-STAR not reported  No reference to a protocol given  The PICO for the COS is not clearly stated  Very broad area for the COS development and a very large number of outcomes included in the final COS. |
| Misscarriage, abortion and stillbirth | | | | |
| Kim et al. (18)  2021 | Interventions to prevent stillbirth  Clinical trials | 50  6  11 | Outcomes relating to the mother:   - Fetal loss, - Onset of and mode of delivery, - Maternal mortality or near miss, - Psychological and social impact on the women - Women's knowledge   Outcomes relating to the baby:   - Timing of stillbirth, - Neonatal mortality, - Gestational age at delivery, - Birthweight, - Congenital anomaly, - NICU/SCBUor other higher-level neonatal care length of stay | Some details form COS-STAR not reported  Not clear how they selected the 50 starting outcomes from a list of 298. Three different consensus meetings were held, some including stakeholders not participating in the delphi survey.  Does not specify the number of non-responders from the first survey.  Not able to access protocol  Criteria for inclusion in COS during, meetings are not clear.  Number of nations included not clear. |
| Fialaet al, (19)  2018 | Pregnancy  First trimester medical termination | NA  NA  5 | - Success - Failure (ongoing pregnancy) - Need for additional treatment (medical or surgical) to complete MToP (missed abortion, incomplete abortion) - Complications   The woman’s request for additional treatment (medical or surgical) | Significant details from COS-STAR not reported  The aim is to article is to standardize the definition of the outcomes  No reference to a protocol given  No Delphi survey |
| Mental Health | | | | |
| Hellberg et al, (20)  2021 | Pregnant women or their partners suffering from perinatal depression  Any type of treatment  Clinical trials | 98  7  9 | - Self-assessed symptoms of depression - Diagnosis of depression by a clinician - Parent to infant bonding - Self-assessed symptoms of anxiety - Quality of life - Satisfaction with intervention - Suicidal thoughts, attempted or committed suicide - Thoughts of harming the baby, including thoughts of extended suicide - Adverse event | Good compliance with COS-STAR |
| Labour and delivery and complications | | | | |
| Gachon et al, (21)  2021 | Women undergoing operative vaginal delivery  mediolateral episiotomy  A French national prospective study | 65 for clinicians and 82 for patients  18 for clinicians and 14 for patients  51 | 63 variables  Outcomes:  Immediate maternal morbidity   - Perineal tear occurrence - Technique used for repairing perineal tear - Type of suture thread used for repairing perineal tear - Perineal hematoma requiring surgical drainage - Perineal infection - Perineal dehiscence - Self-rated degree of satisfaction during childbirth - Maximal pain felt at perineum - Self-rated perineal pain 3 days after delivery - Perineal hemorrhage of more than 500mL - Blood transfusion requirement - Postpartum urinary retention - Mother’s analgesic consumption - Psychological care required after childbirth - Difficulty for mother-child relationship - Difficulty for moving - Self-rated level of understanding about intervention for the delivery   One-year maternal morbidity   - Postnatal anal incontinence - Postnatal urinary incontinence - Postnatal perineal physical therapy - Perineal surgery since childbirth - Any consultation for anal incontinence since childbirth - Any consultation for urinary incontinence since childbirth - Any consultation for perineal pain since childbirth - Any consultation for depression since childbirth - Antidepressants consumption since childbirth - Perineal pain other than dyspareunia - Postnatal dyspareunia - Sexual intercourse since childbirth - Self-rated satisfaction about sexuality - Self-rated health - Depression - In case pf another pregnancy, existence of a wish for a vaginal delivery - In case pf another pregnancy, existence of a wish for a birth project establishment - Existence of another planned pregnancy - Sexual intercourse since childbirth   Immediate neonatal morbidity   - Neonatal cephalic marks - Hematoma on the head (Cephalhematoma, subgaleal hemorrhage) - Analgesics consumption (neonatal pain) - Birthweight - Skull fracture - Humeral (arm) fracture - Neonate admission into intensive care - Apgar score at five minutes - Arterial cord pH - Clavicle fracture - Humeral fracture - Neonate death | Some details form COS-STAR not reported  No protocol for the COS study.  Not sure how the outcomes included in the study were collected.  Separate surveys for clinicians and patients. Not clear how results from the different surveys were combined. Not sure if researchers are part of the process.  A very large number of outcomes included in the final COS |
| Briscoe et al, (22)  2019 | Caesarean deliveries with infectious morbidity outcome  Clinical trials | 511  Outcomes were grouped into 20 primary outcome groups  4  6 | - Endometritis (primary outcome) - Maternal mortality - Wound infection - Wound complications - Febrile morbidity - Neonatal morbidity | Significant details from COS-STAR not reported  No reference to a protocol given  Only includes authors of systematic reviews in the process |
| Dos Santos et al, (23)  2018 | Pregnant women  Induction of labour  Clinical trials | 93 reduced to 77 by combining different outcomes after first survey round  4  28 | Short-term maternal outcomes   - Cardiorespiratory arrest - Damage to internal organs - Death - Haemorrhage - Hysterectomy - Infection - Intensive care admission - Length of hospital stay - Mode of delivery - Need for more than one induction agent - Oxytocin augmentation - Postnatal depression - Pulmonary embolus - Satisfaction with care - Stroke - Time from induction to delivery - Uterine hyperstimulation - Uterine scar dehiscence/rupture   Short-term offspring outcomes   - Admission to the neonatal unit - Birth trauma - Death - Hypoxic ischaemic encephalopathy/need for therapeutic hypothermia - Meconium aspiration syndrome - Need for respiratory support - Infection - Seizures   Long-term maternal outcomes   - Operative pelvic floor repair   Long-term offspring outcomes   - Disability including neurodevelopmental delay | Good compliance with COS-STAR  Only a few patient representatives (3/ 20) present in the workshop  A very large number of outcomes included in the final COS |
| Meher et al, (24)  2018 | Postpartum haemorrhage  Two core outcomes sets presented one for prevention and one for treatment  Clinical trials | Prevention:  161 combined into 35  16  9  Treatment:  97 combined into 31  18  12 | Prevention of postpartum haemorrhage:   - blood loss - shock - maternal death - use of additional uterotonics - blood transfusion - transfer for higher level of care - women’s sense of wellbeing - acceptability and satisfaction with the intervention - breastfeeding - adverse effects   Treatment of postpartum haemorrhage:   - blood loss - shock - coagulopathy - hysterectomy - organ dysfunction - maternal death - blood transfusion - use of additional haemostatic intervention - transfer for higher level of care - women’s sense of wellbeing - acceptability and satisfaction with the intervention - breastfeeding - adverse effects | Good compliance with COS-STAR  Not able to access protocol |
| Fetal / neonatal | | | | |
| Healy et al, (25)  2019 | Fetal growth restriction prevention and treatment | 103  Not enough information provided  22 | - Preeclampsia - Eclampsia - Maternal death - Mode of birth - Fetal stillbirth/livebirth - Gestational age at birth - Preterm birth (delivery at <37 weeks gestation) - Extremely preterm birth (delivery at<28 weeks gestation) - Birthweight - Birthweight <10th percentile - Birthweight <3rd percentile - Need for mechanical ventilation - Bronchopulmonary dysplasia/chronic lung disease - Necrotizing enterocolitis - Neonatal seizures - Hypoxic ischemic encephalopathy - Neonatal death - Childhood cognitive impairment - Motor impairment - Cerebral palsy - Hearing Impairment - Visual Impairment | Good compliance with COS-STAR  A very large number of outcomes included in the final COS |
| Perry et al, (26)  2019 | Twin – twin transfusion syndrome (TTTS) treatments | 71  21  12 | - Live birth - Pregnancy loss (including miscarriage, stillbirth, termination of pregnancy and neonatal mortality) - Subsequent death of a cotwin following single-twin demise at the time of treatment - Recurrence of TTTS - Twin anemia – polycythemiasequence and amniotic band syndrome - Gestational age at delivery - Birthweight - Brain injury syndromes - Ischemic limb injury - Maternal mortality - Admission to Level-2 or -3 care setting - One aspirational outcome - Neurodevelopment at 18–24 months of age | Good compliance with COS-STAR  Does not specify the number of non-responders from the first survey. |
| Townsend et al, (27)  2019 | Selective fetal growth restriction in twins management clinical trails | 96 identified in SR, 56 included in the first round  7  11 | - Live birth - Gestational age at birth - Birth weight - Inter-twin birthweight discordance - Death of surviving twin after death of co-twin - Loss during pregnancy or before final hospital discharge (miscarriage, stillbirth, termination of the pregnancy, neonatal death, perinatal death) - Parental stress - Procedure-related adverse outcome (failure of procedure, procedure to delivery interval, placenta abruption, life threatening haemorrhage, sepsis, maternal death) - Length of stay in hospital (neonatal) - Neurological abnormalities on postnatal imaging - Childhood disability | Good compliance with COS-STAR  Does not specify the number of non-responders from the first survey. |

**BP** =Blood presure; **BMI** = Body Mass Index; **COS** = Core Outcome Set; **COS-STAR** = Core Outcome Set–STAndards for Reporting; **GDM** = Gestational diabetes mellitus; **HbA1c** = Hemoglobin A1c (Långtidsblodsockret); **MToP** = Medical termination of pregnancy; **ITU/HDU** = Intensive care units (/ high dependency units **IQR** = Interquartile range; **NA** = Not applicable; **SR** = Systematic Review **TTTS** = Twin – twin transfusion syndrome

References

1. Bashir M, Syed A, Furuya-Kanamori L, Musa OAH, Mohamed AM, Skarulis M, et al. Core outcomes in gestational diabetes for treatment trials: The Gestational Metabolic Group treatment set. Obesity Science & Practice. 2021;7(3):251-9.

2. Meissner Y, Fischer-Betz R, Andreoli L, Costedoat-Chalumeau N, De Cock D, Dolhain R, et al. EULAR recommendations for a core data set for pregnancy registries in rheumatology. Ann Rheum Dis. 2021;80(1):49-56.

3. Duffy JMN, Cairns AE, Richards‐Doran D, t Hooft J, Gale C, Brown M, et al. A core outcome set for pre-eclampsia research: an international consensus development study. BJOG: An International Journal of Obstetrics & Gynaecology. 2020;127(12):1516-26.

4. Egan AM, Bogdanet D, Griffin TP, Kgosidialwa O, Cervar-Zivkovic M, Dempsey E, et al. A core outcome set for studies of gestational diabetes mellitus prevention and treatment. Diabetologia. 2020;63(6):1120-7.

5. Jansen L, Koot MH, Van't Hooft J, Dean CR, Duffy J, Ganzevoort W, et al. A core outcome set for hyperemesis gravidarum research: an international consensus study. BJOG : an international journal of obstetrics and gynaecology. 2020;127(8):983-92.

6. Bogdanet D, Reddin C, Macken E, Griffin TP, Fhelelboom N, Biesty L, et al. Follow-up at 1 year and beyond of women with gestational diabetes treated with insulin and/or oral glucose-lowering agents: a core outcome set using a Delphi survey. Diabetologia. 2019.

7. Bunch KJ, Allin B, Jolly M, Hardie T, Knight M. Developing a set of consensus indicators to support maternity service quality improvement: using Core Outcome Set methodology including a Delphi process. BJOG: An International Journal Of Obstetrics And Gynaecology. 2018;125(12):1612-8.

8. Nijagal MA, Wissig S, Stowell C, Olson E, Amer-Wahlin I, Bonsel G, et al. Standardized outcome measures for pregnancy and childbirth, an ICHOM proposal. BMC Health Services Research. 2018;18(1):953-.

9. Egan AM, Galjaard S, Maresh MJA, Loeken MR, Napoli A, Anastasiou E, et al. A core outcome set for studies evaluating the effectiveness of prepregnancy care for women with pregestational diabetes. Diabetologia. 2017;60(7):1190-6.

10. Al Wattar BH, Tamilselvan K, Khan R, Kelso A, Sinha A, Pirie AM, et al. Development of a core outcome set for epilepsy in pregnancy (E-CORE): a national multi-stakeholder modified Delphi consensus study. BJOG : an international journal of obstetrics and gynaecology. 2017;124(4):661-7.

11. Rogozinska E, D'Amico MI, Khan KS, Cecatti JG, Teede H, Yeo S, et al. Development of composite outcomes for individual patient data (IPD) meta-analysis on the effects of diet and lifestyle in pregnancy: a Delphi survey. BJOG: An International Journal of Obstetrics & Gynaecology. 2016;123(2):190-8.

12. van ʼt Hooft J, Duffy JMN, Daly M, Williamson PR, Meher S, Thom E, et al. A Core Outcome Set for Evaluation of Interventions to Prevent Preterm Birth. Obstetrics And Gynecology. 2016;127(1):49-58.

13. Fong F, Rogozinska E, Allotey J, Kempley S, Shah DK, Thangaratinam S. Development of maternal and neonatal composite outcomes for trials evaluating management of late-onset pre-eclampsia. Hypertens Pregnancy. 2014;33(2):115-31.

14. Saldanha IJ, Wilson LM, Bennett WL, Nicholson WK, Robinson KA. Development and pilot test of a process to identify research needs from a systematic review. Journal of Clinical Epidemiology. 2013;66(5):538-45.

15. Bennett WL, Robinson KA, Saldanha IJ, Wilson LM, Nicholson WK. High priority research needs for gestational diabetes mellitus. Journal of women's health (2002). 2012;21(9):925-32.

16. Mehra H, Thangaratinam S. Prioritisation of outcomes in the evaluation of weight management interventions in pregnancy: A DELPHI survey. Archives of Disease in Childhood: Fetal and Neonatal Edition. 2012;97:A38.

17. Devane D, Begley CM, Clarke M, Horey D, Oboyle C. Evaluating maternity care: a core set of outcome measures. Birth (Berkeley, Calif). 2007;34(2):164-72.

18. Kim BV, Aromataris EC, Middleton P, Townsend R, Thangaratinam S, Duffy JMN, et al. Development of a core outcome set for interventions to prevent stillbirth. Aust N Z J Obstet Gynaecol. 2021.

19. Fiala C, Cameron S, Bombas T, Parachini M, Agostini A, Lertxundi R, et al. Outcome of first trimester medical termination of pregnancy: definitions and management. European Journal of Contraception & Reproductive Health Care. 2018;23(6):451-7.

20. Hellberg C, Osterberg M, Jonsson AK, Fundell S, Tronnberg F, Jonsson M, et al. Important research outcomes for treatment studies of perinatal depression: systematic overview and development of a core outcome set. BJOG : an international journal of obstetrics and gynaecology. 2021.

21. Gachon B, Schmitz T, Artzner F, Parant O, De Tayrac R, Ducarme G, et al. A core outcome set development for a French national prospective study about the effect of mediolateral episiotomy on obstetric anal sphincter injury during operative vaginal delivery (INSTRUMODA). BMC pregnancy and childbirth. 2021;21(1):251.

22. Briscoe KE, Haas DM. Developing a Core Outcome Set for Cesarean Delivery Maternal Infectious Morbidity Outcomes. American journal of perinatology. 2019.

23. Dos Santos F, Drymiotou S, Antequera Martin A, Mol BW, Gale C, Devane D, et al. Development of a core outcome set for trials on induction of labour: an international multistakeholder Delphi study. BJOG : an international journal of obstetrics and gynaecology. 2018;125(13):1673-80.

24. Meher S, Cuthbert A, Kirkham JJ, Williamson P, Abalos E, Aflaifel N, et al. Core outcome sets for prevention and treatment of postpartum haemorrhage: an international Delphi consensus study. BJOG: An International Journal Of Obstetrics And Gynaecology. 2019;126(1):83-93.

25. Healy P, Gordijn SJ, Ganzevoort W, Beune IM, Baschat A, Khalil A, et al. A Core Outcome Set for the prevention and treatment of fetal GROwth restriction: deVeloping Endpoints: the COSGROVE study. Am J Obstet Gynecol. 2019;221(4):339.e1-.e10.

26. Perry H, Duffy JMN, Reed K, Baschat A, Deprest J, Hecher K, et al. Core outcome set for research studies evaluating treatments for twin-twin transfusion syndrome. Ultrasound Obstet Gynecol. 2019;54(2):255-61.

27. Townsend R, Duffy JMN, Sileo F, Perry H, Ganzevoort W, Reed K, et al. A core outcome set for studies investigating the management of selective fetal growth restriction in twins. Ultrasound Obstet Gynecol. 2019.
