# Supplementary material for: Core Outcome Sets (COS) related to pregnancy and childbirth: a systematic review
Source: BMC Pregnancy Childbirth. 2021 Oct 9;21:691. doi: 10.1186/s12884-021-04164-y (PMC8501579; doi:10.1186/s12884-021-04164-y)
Supplement: Supplementary file 4 — Additional file 4: Table S1. Excluded studies. [file 12884_2021_4164_MOESM4_ESM.docx]

**Table S1 Excluded studies**

| **Study** | **Reason for exclusion** |
| --- | --- |
| A Core Outcome Set for Very Preterm Birth?, http://www.comet-initiative.org/studies/details/256 | Not a Core Outcome Set |
| Allin B, Bradnock T, Kenny S, Walker G, Knight M. NETS1HD: study protocol for development of a core outcome set for use in determining the overall success of Hirschsprung's disease treatment. Trials 2016;17:1-7. | Outside prespecified population |
| Allin B, Ross A, Marven S, Hall NJ, Knight M, J Hall N. Development of a core outcome set for use in determining the overall success of gastroschisis treatment. Trials 2016;17:1-7. | Outside prespecified population |
| An International Urogynecological Association (IUGA)/International Continence Society (ICS) joint report on the terminology for reporting outcomes of surgical procedures for pelvic organ prolapse, http://www.comet-initiative.org/studies/details/525 | Not a Core Outcome Set |
| Bakhbakhi D, Burden C, Fraser A, Hinton L, Duffy J, Redshaw M, et al. Development of a core outcome set and identification of outcome measurement tools for interventions after stillbirth. J Evid Based Med 2019;12:10. | Duplication with a published protocol/registration in the COMET initiative database/or finished COS article |
| Bakhbakhi D, Duffy JMN, Hinton L, Burden C, Main B, Downe S, et al. Development of a core outcome set for interventions after stillbirth. Int J Gynaecol Obstet 2018;143:235. | Duplication with a published protocol/registration in the COMET initiative database/or finished COS article |
| Bannatyne AJ, Hughes R, Stapleton P, Watt B, MacKenzie-Shalders K. Signs and symptoms of disordered eating in pregnancy: a Delphi consensus study. BMC Pregnancy Childbirth 2018;18:262-262. | Not a Core Outcome Set |
| Bogdanet D, Egan A, Fhelelboom N, Biesty L, Thangaratinam S, Dempsey E, et al. Metabolic follow-up at one year and beyond of women with gestational diabetes treated with insulin and/or oral hypoglycaemic agents: study protocol for the identification of a core outcomes set using a Delphi survey. Trials 2019;20:9. | Duplication with a published protocol/registration in the COMET initiative database/or finished COS article |
| Bunch K, Allin B, Knight M. Authors' reply re: Developing a set of consensus indicators to support maternity service quality improvement: using Core Outcome Set methodology including a Delphi process. BJOG 2019;126:130-130. | Not a Core Outcome Set |
| Butler MM, Brosnan MC, Drennan J, Feeney P, Gavigan O, Kington M, et al. Evaluating midwifery-led antenatal care: using a programme logic model to identify relevant outcomes. Midwifery 2014;30:e34-41. | Not a Core Outcome Set |
| Clayton AH, Dennerstein L, Fisher WA, Kingsberg SA, Perelman MA, Pyke RE. Standards for clinical trials in sexual dysfunction in women: research designs and outcomes assessment. J Sex Med 2010;7:541-60. | Outside prespecified population |
| Core outcomes for twin anaemia polycythaemia sequence in Twin pregnancies, https://www.comet-initiative.org/studies/details/1139 | Withdrawn |
| Dadouch R, Faheim M, Juando-Prats C, Parsons J, D'Souza R. Development of a core outcome set for studies on obesity in pregnant patients (COSSOPP): A study protocol. J Evid Based Med 2019;12:19. | Duplication with a published protocol/registration in the COMET initiative database/or finished COS article |
| Dadouch R, Rosen C, Parsons J, D'Souza R. Obesity in pregnancy patient-reported outcomes: A qualitative study. J Evid Based Med 2019;12:18-9. | Not a Core Outcome Set |
| Developing a core outcome set (COS) for intrapartum fetal assessment, https://www.comet-initiative.org/studies/details/741 | Withdrawn |
| D'Souza R, Hall C, Siu S, Sermer M, Silversides C. Patient-reported outcomes in pregnancy and heart disease: A qualitative study. J Evid Based Med 2019;12:20. | Not a Core Outcome Set |
| D'Souza R, Thurman R, Sermer M, Siu S, Duffy J, Silversides C. Developing a core outcome set for pregnant women with cardiac disease. J Evid Based Med 2017;10:24-5. | Duplication with a published protocol/registration in the COMET initiative database/or finished COS article |
| D'Souza RD, Thomas H, Wuebbolt D, Nguyen V, Sermer M, Krahn M, et al. Preferences of pregnant women with cardiac disease for combined maternal-fetal health states. J Evid Based Med 2017;10:22-3. | Not a Core Outcome Set |
| Duffy J, Rolph R, Gale C, Hirsch M, Khan KS, Ziebland S, et al. Core outcome sets in women's and newborn health: a systematic review. BJOG 2017;124:1481-9. | Not a Core Outcome Set |
| Duffy J, Thompson T, Hinton L, Salinas M, McManus RJ, Ziebland S. What outcomes should researchers select, collect and report in pre-eclampsia research? A qualitative study exploring the views of women with lived experience of pre-eclampsia. BJOG 2019;126:637-46. | Not a Core Outcome Set |
| Duffy JMN, Ziebland S, von Dadelszen P, McManus RJ. Tackling poorly selected, collected, and reported outcomes in obstetrics and gynecology research. American journal of obstetrics and gynecology 2019;220:71.e1-71.e4. | Not a Core Outcome Set |
| Egan AM, Smith V, Devane D, Dunne FP. Effectiveness of prepregnancy care for women with pregestational diabetes mellitus: protocol for a systematic review of the literature and identification of a core outcomes set using a Delphi survey. Trials 2015;16:356-356. | Protocol for a completed COS |
| Einerson BD, Einerson BD. Getting it right: core outcome sets in quality improvement research. BJOG 2018;125:1619-1619. | Not a Core Outcome Set |
| Fejzo MS, Fejzo MS. A core outcome set for hyperemesis gravidarum studies is a small step in the right direction. BJOG: An International Journal of Obstetrics & Gynaecology 2020;127:993-993. | Not a Core Outcome Set |
| Fong F, Rogozinska E, Allotey J, Kempley S, Shah D, Thangaratinam S. Identification of clinically important components of maternal and neonatal composite outcomes to assess the effect of timing of delivery in women with mild to moderate pre-eclampsia at 34 to 37 weeks. Arch Dis Child Fetal Neonatal Ed 2013;98. | Duplication with a published protocol/registration in the COMET initiative database/or finished COS article |
| Hall C, D'Souza RD. Patients and Health Care Providers Identify Important Outcomes for Research on Pregnancy and Heart Disease. CJC open 2020;2:454-461. | Not a Core Outcome Set |
| Hooft J, van 't Hooft J. A core outcome set for evaluation of interventions to prevent preterm birth: summary for CROWN. BJOG 2016;123:666-666. | Not a Core Outcome Set |
| Kelly LE, Jansson LM, Moulsdale W, Pereira J, Simpson S, Guttman A, et al. A core outcome set for neonatal abstinence syndrome: study protocol for a systematic review, parent interviews and a Delphi survey. Trials [Electronic Resource] 2016;17:536. | Outside prespecified population |
| Khan K. The Core Outcomes in Women's Health (CROWN) Initiative: Journal Editors Invite Researchers to Develop Core Outcomes in Women's Health. Gynecologic And Obstetric Investigation 2015;80:1-2. | Not a Core Outcome Set |
| Khan K. The CROWN Initiative: journal editors invite researchers to develop core outcomes in women's health. BJOG 2016;123:103-4. | Not a Core Outcome Set |
| Khan K. The CROWN Initiative: journal editors invite researchers to develop core outcomes in women's health. Hypertens Pregnancy 2014;33:261-4. | Not a Core Outcome Set |
| Khan K. The CROWN Initiative: journal editors invite researchers to develop core outcomes in women's health. Journal Of Perinatal Medicine 2014;42:543-4. | Not a Core Outcome Set |
| Khan K. The CROWN initiative: journal editors invite researchers to develop core outcomes in women's health. Obstetrics And Gynecology 2014;124:487-8. | Not a Core Outcome Set |
| Killeen S, O'Brien E, Geraghty A, McAuliffe F. Maternal nutrition in pregnancy: A protocol for the development of a core outcome set. Obes Facts 2019;12:222. | Duplication with a published protocol/registration in the COMET initiative database/or finished COS article |
| Killeen SL, O'Brien EC, Geraghty AA, McAuliffe F. A protocol for the development of a core outcome set for research on maternal nutrition during pregnancy. BJOG 2019;126:46. | Duplication with a published protocol/registration in the COMET initiative database/or finished COS article |
| King A, Leider H, Herman D, Malinowski AK, D'Souza R. Patient- and Health-Care-Provider-Reported Outcomes to Consider in Research on Pregnancy-Associated Venous Thromboembolism. Thrombosis and haemostasis 2021. | Not a Core Outcome Set |
| Kuo J, Petrie KJ, Alsweiler JM. Prioritising long-term outcomes for babies born preterm: The hip survey. J Paediatr Child Health 2019;55:83. | Outside prespecified population |
| Lakhanpaul M, Irish C, Jarvis R, Edbrooke-Childs J, Deighton J, Franklin M, et al. A shared outcome perinatal mental health value scorecard to support health visitors to improve outcomes for children 0-5 years and their families. Arch Dis Child 2016;101:A91. | Outside prespecified population |
| Lumsden MA. Will the development of a core outcome set on prevention and treatment of postpartum haemorrhage add value to research or clinical care? BJOG 2019;126:95-95. | Not a Core Outcome Set |
| Malinowski AK, Daru J, D'Souza R, Shehata N. Constructing a core outcome set for iron deficiency and iron deficiency anemia in pregnancy and postpartum. J Evid Based Med 2017;10:37. | Duplication with a published protocol/registration in the COMET initiative database/or finished COS article |
| Malinowski AK, Shehata N, D'Souza R. Constructing a core outcome set for immune thrombocytopenia in pregnancy. J Evid Based Med 2017;10:38. | Duplication with a published protocol/registration in the COMET initiative database/or finished COS article |
| Malinowski AK, Shehata N, D'Souza R. Constructing a core outcome set for venous thromboembolism in pregnancy. J Evid Based Med 2017;10:37-8. | Duplication with a published protocol/registration in the COMET initiative database/or finished COS article |
| Mann S, Pratt S, Gluck P, Nielsen P, Risser D, Greenberg P, et al. Assessing quality obstetrical care: development of standardized measures Jt Comm J Qual Patient Saf 2006;32:497-505. | Not a Core Outcome Set |
| Mawer G. Core outcomes for studies of pregnancy with epilepsy. BJOG 2017;124:668-668. | Not a Core Outcome Set |
| McMaster-Fay RA, Hyett JA. Fetal growth restriction: a core set of outcome endpoints. American journal of obstetrics and gynecology 2020;222:390. | Not a Core Outcome Set |
| Medley N, Alfirevic Z, Caldwell DM, Dias S, Dowswell T, Keeney E, et al. Outcomes reported in trials of methods for the induction of labour. Trials 2015;16. | Not a Core Outcome Set |
| Myatt L, Redman CW, Staff AC, Hansson S, Wilson ML, Laivuori H, et al. Strategy for standardization of preeclampsia research study design. Hypertension 2014;63:1293-301. | Not a Core Outcome Set |
| Nguyen V, Wuebbolt D, Thomas H, Shehata N, Krahn M, D'Souza R. Iron deficiency anemia in pregnancy and treatment options: A patient-preference study. J Evid Based Med 2017;10:24. | Not a Core Outcome Set |
| Outcomes Important to Patients Public and Practitioners (OMIPPP): Breastfeeding, http://www.comet-initiative.org/studies/details/662 | Not a Core Outcome Set |
| Patient Preferences and Experiences in Hyperemesis Gravidarum Treatment: A Qualitative Study, http://www.comet-initiative.org/studies/details/1334 | Not a Core Outcome Set |
| Perry H, Duffy JMN, Umadia O, Khalil A. Outcome reporting across randomized trials and observational studies evaluating treatments for twin-twin transfusion syndrome: systematic review. Ultrasound Obstet Gynecol 2018;52:577-85. | Not a Core Outcome Set |
| Petersen R, Nijagal M, Wissig S, Stowell C, Franx A. Defining an international standard set of outcomes measures for maternity care: Consensus of the international consortium of health outcomes measurement pregnancy & childbirth working group. Aust N Z J Obstet Gynaecol 2016;56:51-2. | Duplication with a published protocol/registration in the COMET initiative database/or finished COS article |
| Progressing towards core outcomes for maternal and perinatal clinical trials and reviews. J Paediatr Child Health 2017;53:91-91. | Not a Core Outcome Set |
| Rahn DD, Abed H, Sung VW, Matteson KA, Rogers RG, Morrill MY, et al. Systematic review highlights difficulty interpreting diverse clinical outcomes in abnormal uterine bleeding trials. J Clin Epidemiol 2011;64:293-300. | Outside prespecified population |
| Read SK, Jibril A, Tongo O, Akindolire A, Abdulkadir I, Nabwera H, et al. Parents' perceptions of core outcomes in neonatal research in two Nigerian neonatal units. BMJ paediatrics open 2020;4:e000669. | Not a Core Outcome Set |
| Ricciardi P, Haydar A. Re: Developing a set of consensus indicators to support maternity service quality improvement: using Core Outcome Set methodology including a Delphi process: The Robson 10-groups classification system - all groups are needed. BJOG 2019;126:129-30. | Not a Core Outcome Set |
| Rogers J, Spink M, Magrill A, Burgess K, Agius M. Evaluation of a Specialised Counselling Service for Perinatal Bereavement. Psychiatr Danub 2015;27:S482-S5. | Not a Core Outcome Set |
| Rosen C, Dadouch R, Parsons J, D'Souza R. The comparison of outcomes reported by healthcare professionals and patients on the management of obesity in pregnancy. J Evid Based Med 2019;12:9. | Not a Core Outcome Set |
| Rysavy MA, Marlow N, Doyle LW, Tyson JE, Serenius F, Iams JD, et al. Reporting Outcomes of Extremely Preterm Births. Pediatrics 2016;138. | Not a Core Outcome Set |
| Say RE, Thomson RG, Robson SC. Making high quality decisions in pregnancy: How should interventions which aim to improve decision quality be evaluated? Arch Dis Child Fetal Neonatal Ed Edition 2012;97:A114-A5. | Not a Core Outcome Set |
| Schaap T, Bloemenkamp K, Deneux-Tharaux C, Knight M, Langhoff-Roos J, Sullivan E, et al. Defining definitions: a Delphi study to develop a core outcome set for conditions of severe maternal morbidity. BJOG 2019;126:394-401. | Not a Core Outcome Set |
| Signs and symptoms of disordered eating in pregnancy: a Delphi consensus study, http://www.comet-initiative.org/studies/details/1339 | Not a Core Outcome Set |
| Slavin V, Gamble J, Creedy DK, Fenwick J. “Coming of Age”: assessing the feasibility of using a core set of value-based health outcomes for pregnancy and childbirth. Women Birth 2018;31:S29-S30. | Not a Core Outcome Set |
| Smith CA, Betts D. The practice of acupuncture and moxibustion to promote cephalic version for women with a breech presentation: implications for clinical practice and research. Complement Ther Med 2014;22:75-80. | Not a Core Outcome Set |
| Son M. Core outcome set for induction of labour trials: what's the expectation? BJOG 2018;125:1681-1681. | Not a Core Outcome Set |
| Spink M, Magrill A, Burgess K, Rogers J, Agius M. Petals: An Assessment of the Outcomes of a Service for Bereavement during Childbirth. Psychiatr Danub 2014;26:211-21. | Not a Core Outcome Set |
| Standards for clinical trials in sexual dysfunction in women: research designs and outcomes assessment, http://www.comet-initiative.org/studies/details/411 | Outside prespecified population |
| Stevens G, Donnelly KZ, Theiler RN, Washburn H, Woodhams EJ, Lindahl V, et al. (Family) planning ahead: User-centered design of the birth control after pregnancy patient decision aid and protocol for delivery and evaluation. Contraception 2017;96:289. | Outside prespecified population |
| Strategy for standardization of preeclampsia research study design, http://www.comet-initiative.org/studies/details/610 | Not a Core Outcome Set |
| The core outcomes in women's health (CROWN) initiative. Eur J Obstet Gynecol Reprod Biol 2014;180:A1-A2. | Not a Core Outcome Set |
| The practice of acupuncture and moxibustion to promote cephalic version for women with a breech presentation: implications for clinical practice and research, http://www.comet-initiative.org/studies/details/728 | Not a Core Outcome Set |
| Toozs-Hobson P, Freeman R, Barber M, Maher C, Haylen B, Athanasiou S, et al. An International Urogynecological Association (IUGA)/International Continence Society (ICS) joint report on the terminology for reporting outcomes of surgical procedures for pelvic organ prolapse. Neurourol Urodyn 2012;31:415-21. | Not a Core Outcome Set |
| van der Aa JE, Tancredi A, Goverde AJ, Velebil P, Feyereisl J, Benedetto C, et al. What European gynaecologists need to master: Consensus on medical expertise outcomes of pan-European postgraduate training in obstetrics & gynaecology. Eur J Obstet Gynecol Reprod Biol 2017;216:143-52. | Not a Core Outcome Set |
| van 't Hooft J, Alfirevic Z, Asztalos EV, Biggio JR, Dugoff L, Hoffman M, et al. CROWN initiative and preterm birth prevention: researchers and editors commit to implement core outcome sets. BJOG 2018;125:8-11. | Not a Core Outcome Set |
| van 't Hooft J. A core outcome set for evaluation of interventions to prevent preterm birth: summary for CROWN. BJOG 2016;123:107-107. | Not a Core Outcome Set |
| van Vliet R, Bink M, Polman J, Suntharan A, Grooten I, Zwolsman SE, et al. Patient Preferences and Experiences in Hyperemesis Gravidarum Treatment: A Qualitative Study. J Pregnancy 2018;2018:5378502. | Not a Core Outcome Set |
| Van't Hooft J, Duffy JMN, Saade GR, Alfirevic Z, Meher S, Mol BWJ, et al. Core outcomes set for studies on primary prevention of preterm birth. Trials 2015;16. | Duplication with a published protocol/registration in the COMET initiative database/or finished COS article |
| Van't Hooft J, Khan KS. P-hacking can be avoided with core outcome sets: preterm birth research is ready to take this leap. BJOG 2017;124:1017-1017. | Not a Core Outcome Set |
| Webbe J, Brunton G, Afonso E, Latour JM, Gale C. The importance of core outcome sets and developing one for neonatal care. Infant 2017;13:70-2. | Outside prespecified population |
| Webbe J, Brunton G, Ali S, Duffy JM, Modi N, Gale C. Developing, implementing and disseminating a core outcome set for neonatal medicine. BMJ Paediatr Open 2017;1:e000048. | Outside prespecified population |
| Webbe J, Brunton G, Ali S, Wann L, Modi N, Gale C. Using existing data sources to extract parent and patient outcomes for a neonatal core outcome set. J Evid Based Med 2017;10:10-11. | Outside prespecified population |
| What outcomes should researchers select, collect and report in pre-eclampsia research? A qualitative study exploring the views of women with lived experience of pre-eclampsia, http://www.comet-initiative.org/studies/details/1327 | Not a Core Outcome Set |
| Wiegers TA, Keirse MJ, Berghs GA, van der Zee J. An approach to measuring quality of midwifery care. J Clin Epidemiol 1996;49:319-25. | Not a Core Outcome Set |
| Williamson P. Core outcome sets will improve the quality of obstetrics research. BJOG 2014;121:1196. | Not a Core Outcome Set |
| Wu N, O'Reilly S, Nielsen KK, Maindal HT, Dasgupta K. Core outcome set for diabetes after pregnancy prevention across the life span: international Delphi study. BMJ open diabetes research & care 2020;8. | Outside prespecified population |
