# Supplementary material for: Core Outcome Sets (COS) related to pregnancy and childbirth: a systematic review
Source: BMC Pregnancy Childbirth. 2021 Oct 9;21:691. doi: 10.1186/s12884-021-04164-y (PMC8501579; doi:10.1186/s12884-021-04164-y)
Supplement: Supplementary file 5 — Additional file 5: Table S2. Ongoing COS studies. [file 12884_2021_4164_MOESM5_ESM.docx]

**Table S2 Ongoing COS studies**

| Reference  First author  Year | Population | Stakeholders to be included in the workgroup | Methods | Compliance with COS-STAR |
| --- | --- | --- | --- | --- |
| (1)  PI: Dr J. Oliver Daly  (registered in COMET database 959) | Prevention and treatment of obstetric anal sphincter injuries  COS for clinical trials or clinical research and COS for practice | - Clinical experts - Consumers (patients) - Epidemiologists - Journal Editors - Patient/support group representatives | - Systematic review to identify outcome measures - International survey of stakeholders to review identified outcomes and other outcomes identified by stakeholders - Delphi methodology to achieve a consensus | Not enough information |
| (2)  PI: A/Prof Rae-Chi Huang  (registered in COMET database 784) | Interventions for overweight women before pregnancy and in the first trimester of pregnancy  COS for clinical trials or clinical research COS for practice | - Clinical experts - Consumers (patients) - Economists - Epidemiologists - Families - Researchers - Service providers Statisticians | - Systematic review to identify outcome measures - A Delphi process until reduction of the list of identified outcomes to ten or less or on completion of the second round - Consensus face-to-face meeting | Not enough information |
| (3)  D'Souza et al 2020  (registered in COMET database 834) | Therapeutic interventions for pregnant women with cardiac disease  COS for clinical trials or clinical research, COS for practice | - Clinical experts - Consumers (patients) - Guideline developers - Journal editors - Researchers - Service providers - Statisticians | - Systematic review to identify outcome measures - Two round Delphi survey to achieve a consensus - Consensus face-to-face meeting - Recommendations for outcome measures (measurement/how | Not enough information |
| (4)  PI: Dr. Ann Kinga Malinowski  (registered in COMET database 836) | Identification, assessment and management of iron deficiency and iron deficiency anaemia in pregnancy and postpartum  COS for clinical trials or clinical research | - Clinical experts - Consumers (patients) - Researchers | - Systematic review to identify outcome measures - Two round Delphi survey to achieve a consensus | Not enough information |
| (5)  King et al 2020  (registered in COMET database 839) | Identification, assessment and management of venous thromboembolism in pregnancy  COS for clinical trials or clinical research | - Clinical experts - Consumers (patients) - Researchers | - Systematic review to identify outcome measures - Two round Delphi survey to achieve a consensus | Not enough information |
| (6)  PI: Dr. Ann Kinga Malinowski  (registered in COMET database 840) | The evolution and management of immune thrombocytopenia in pregnancy  COS for clinical trials or clinical research | - Clinical experts - Researchers | - Systematic review to identify outcome measures - Two round Delphi survey to achieve a consensus | Not enough information |
| (7)  PI: Dr R. Katie Morris  (registered in COMET database 844) | Research in multiple pregnancies, irrespective of chorionicity  COS for clinical trials or clinical research | - Clinical experts - Consumers (patients) - Families - Patient/support group representatives - Researchers | - Systematic review to identify outcome measures - Delphi survey - Consensus face-to-face meeting | Not enough information |
| (8)  prof. dr. F. Amant  (registered in COMET database 1098) | Diagnostic and therapeutic management pregnancies complicated by cancer  COS for clinical trials or clinical research and COS for practice | - Clinical experts - Consumers (caregivers) - Consumers (patients) - Patient/ support group representatives | - Systematic review to identify outcome measures - Delphi survey - If necessary, consensus meeting | Not enough information |
| (9)  D'Souza et al 2020  (registered in COMET database 1117) | Antenatal diagnosis and management pregnant women with vasa previa  COS for clinical trials or clinical research COS for practice | - Charities - Clinical experts - Conference participants - Consumers (caregivers) - Consumers (patients) - Epidemiologists - Families - Methodologists - Patient/support group representatives - Policy makers - Researchers | - Systematic review to identify outcome measures - Qualitative patient interviews to identify potential core outcomes. - Delphi survey - If necessary, consensus face-to-face meeting - Determining How Core Outcomes Should be Measured | Not enough information |
| (10)  PI: Susan O’Rinn  (registered in COMET database 1127) | Screening, diagnosing, and managing of invasive placentation  COS for clinical trials or clinical research | - Clinical experts - Consumers (caregivers) - Consumers (patients) - Journal editors - Policy makers - Researchers - Service providers - Service users | - Systematic review to identify outcome measures - Focus Groups to identify important outcomes from the perspectives of patients - Delphi survey - Consensus face-to-face meeting | Not enough information |
| (11)  Altoukhi et al 2020  (registered in COMET database 1187) | Fetal Interventions of myelomeningocele (spina bifida)  COS for clinical trials or clinical research COS for practice | - Clinical experts - Consumers (caregivers) - Consumers (patients) - Families - Patient/ support group representatives - Researchers | - Systematic review to identify outcome measures - Delphi survey - Consensus face-to-face meeting | Not enough information |
| (12)  PI: Dr. Ann Kinga Malinowski  (registered in COMET database 1190) | Management of sickle cell disease in pregnancy  COS for clinical trials or clinical research | - Clinical experts - Consumers (patients) - Methodologists - Patient/ support group representatives - Pharmaceutical industry representatives - Researchers - Service providers - Service users | - Systematic review to identify outcome measures - Delphi survey - Consensus face-to-face meeting | Not enough information |
| (13)  Killeen et al 2019  (registered in COMET database 1273) | Maternal nutrition during pregnancy  COS for clinical trials or clinical research COS for practice | - Clinical experts - Consumers (patients) - Researchers | - Systematic review to identify outcome measures - Delphi survey - Steering group meeting | Not enough information |
| (14)  Vergote et al 2021  (registered in COMET database 1296) | Prenatal interventions for congenital diaphragmatic hernia  COS for clinical trials or clinical research | - Clinical experts - Patient/support group representatives - Researchers | - Systematic review to identify outcome measures - Delphi survey - Consensus face-to-face meeting - Determining how core outcomes should be measured | Not enough information |
| (15)  Doumouchtsis et al 2020  (registered in COMET database 981) | Treatment of female pelvic floor disorders (urinary incontinence, pelvic organ prolapse, childbirth perineal trauma and pelvic pain syndromes)  COS for clinical trials or clinical research COS for practice | - Charities - Clinical experts - Consumers (caregivers) - Consumers (patients) - Governmental agencies - Patient/ support group representatives - Pharmaceutical industry representatives - Policy makers Researchers - Service commissioners - Service providers Service users | - Systematic review to identify outcome measures - Qualitative patient interviews to identify potential core outcomes. - Delphi survey - Consensus face-to-face meeting - Determining how core outcomes should be measured | Not enough information |
| (16)  Charlene Sackitey  (registered in COMET database 1082) | Treatment of rectovaginal fistula  COS for clinical trials or clinical research COS for practice | - Charities - Clinical experts - Conference participants - Consumers (caregivers) - Consumers (patients) - Patient/support group representatives - Researchers | - Systematic review to identify outcome measures - Qualitative patient interviews to identify potential core outcomes - Delphi survey - Consensus face-to-face meeting | Not enough information |
| (17)  PI: Dr Danya Bakhbakhi  (registered in COMET database775) | Interventions for prevention of stillbirth and care after stillbirth  COS for clinical trials or clinical research | Unknown | - Systematic review to identify outcome measures - Qualitative patient interviews to identify potential core outcomes - Delphi survey - Consensus face-to-face meeting | Not enough information |
| (18)  Dr Alexander Heazell  (registered in COMET database 928) | Interventions for the detection and management of reduced fetal movements  COS for clinical trials or clinical research | - Clinical experts - Researchers - Service users | - Systematic review to identify outcome measures - Delphi survey - Consensus face-to-face meeting | Not enough information |
| (19)  PI: Dr Stephen O'Brien  (registered in COMET database 1069) | Examining outcomes resulting from operative vaginal birth  COS for clinical trials or clinical research COS for practice | - Charities - Clinical experts - Families - Journal editors - Patient/ support group representatives - Researchers - Service commissioners - Service providers - Service users - Statisticians | - Systematic review to identify outcome measures - Qualitative patient interviews to identify potential core outcomes - Delphi survey - Consensus face-to-face meeting | Not enough information |
| (20)  Christine East  (registered in COMET database 1028) | Intervention to support women and infants to breastfeed.  COS for clinical trials or clinical research | - Clinical experts - Consumers (caregivers) - Researchers | - Systematic review to identify outcome measures - Delphi survey | Not enough information |
| (21)  PI: Dr. Rohan D’Souza  (registered in COMET database 1154) | Maternal and fetal Composite Adverse  Obstetric Outcomes  COS for clinical trials or clinical research | - Clinical experts - Conference participants - Consumers (caregivers) - Consumers (patients) - Epidemiologists - Families - Policy makers - Researchers - Service providers - Service users - Statisticians | - Systematic review to identify outcome measures - Qualitative patient interviews to identify potential core outcomes - Delphi survey - Consensus face-to-face meeting - Determining how core outcomes should be measured | Not enough information |
| (22)  Dadouch et al 2018  (registered in COMET database 939) | Antenatal and peripartum intervention for obesity  COS for clinical trials | - Clinical experts - Conference participants - Consumers (caregivers) - Consumers (patients) - Epidemiologists - Families - Guideline developers - Policy makers - Researchers - Service providers - Service users - Statisticians - Trialists | - Systematic review to identify outcome measures - Qualitative patient interviews to identify potential core outcomes - Two-round Delphi survey - Consensus face-to-face meeting - Determining how core outcomes should be measured | Yes |
| (23)  Smith et al 2017  (registered in COMET database 673) | What constitutes positive health and wellbeing (salutogenesis) in maternity care  COS for clinical trials and measuring in daily intrapartum clinical care. | - Clinical experts - Consumers (patients) - Researchers - Service users | - Three-round Delphi survey | Yes |
| (24)  Smith et al 2017  (registered in COMET database 679 and 816) | Prevention and treatment of miscarriage  COS for clinical trials | - Clinical experts - Consumers (patients) - Families - Patient/ support group representatives | - Systematic review to identify outcome measures - Qualitative patient interviews to identify potential core outcomes - Three-round Delphi survey - Consensus face-to-face meeting | Yes |
| (25)  Whitehouse et al 2017  (registered in COMET database 779) | Medical and surgical abortion  COS for clinical trials | - Clinical experts - Consumers (patients) - Epidemiologists - Governmental agencies - Service providers | - Systematic review to identify outcome measures - Qualitative patient interviews to identify potential core outcomes - Two-round Delphi survey - Consensus face-to-face meeting | Yes |
| (26)  Viau-Lapointe et al 2018  (registered in COMET database 916) | Research on critically ill obstetric patients  COS for clinical trials or clinical research  COS for practice | - Clinical experts - Consumers (caregivers) - Consumers (patients) - Families - Journal editors - Researchers - Service providers | - Systematic review to identify outcome measures - Qualitative patient interviews to identify potential core outcomes - Two-round Delphi survey - Virtual consensus meeting | Yes |
| (27)  Prins et al 2018  (registered in COMET database 1004) | Immune modulation interventions as preventive or therapeutic strategies for pregnancy complications  COS for clinical trials or clinical research  COS for practice | - Clinical experts - Consumers (caregivers) - Consumers (patients) - Patient/support group representatives - Researchers | - Systematic review to identify outcome measures - Three-round Delphi survey - Consensus face-to-face meeting | Yes |
| (28)  Viau-Lapointe et al 2018  Conference abstract | Pregnant women requiring mechanical ventilation  COS for clinical trials | - International group of patients and members of the public | - Systematic review to identify outcome measures - Delphi survey | Not enough information  Conference abstract |
| (29)  Sankaran et al 2015  Conference abstract | Obstetric antiphospholipid antibody syndrome (OAPS), the need for low-molecular weight heparin (LMWH for pregnant women  COS for clinical trials | - Clinicians (rheumatology, obstetrics, maternal-fetal medicine, fertility and haematologists) patients | - Systematic review to identify outcome measures - Delphi survey | Not enough information  Conference abstract |
| (30)  Prof Ben W. Mol  (registered in COMET database 1492) | Ectopic pregnancy  COS for clinical trials or clinical research | - Clinical experts - Patient/ support group representatives - Researchers - Service providers | - Systematic review to identify outcome measures - Delphi survey - Consensus face-to-face meeting - Recommendations for outcome measures (measurement/how) | Not enough information |
| (31)  PI: Professor Tom Bourne,  (registered in COMET database 1763) | Psychopathology following early pregnancy loss  COS for clinical trials or clinical research  COS for practice | - Charities - Clinical experts - Consumers (patients) - Families - Patient/ support group representatives - Researchers | - Systematic review to identify outcome measures - Focus groups or interviews - Delphi process - Consensus meeting | Not enough information |
| (32)  PI: Dr Shawn Walker,  (registered in COMET database 1749) | Breech presentation  COS for clinical trials or clinical research  COS for practice | - Clinical experts - Economists - Patient/ support group representatives - Researchers - Service commissioners - Service users - Statisticians | - Systematic review - Focus groups - Delphi process - Consensus meeting | Not enough information |
| (33)  PI: Pervez Sultan  (registered in COMET database 1728) | Enhanced recovery after cesarean delivery (ERAC)  COS for clinical trials or clinical research | - Clinical experts | - Systematic review - Delphi process - Semi-structured discussion | Not enough information |
| (34)  (registered in COMET database 1724) | Pregnant women with pre-existing multimorbidity  COS for clinical trials or clinical research | - Charities - Clinical experts - Consumers (caregivers) - Consumers (patients) - Patient/ support group representatives - Researchers - Service users | - Literature review - Focus group(s) - Delphi process - Consensus meeting | Not enough information |
| (35)  PI: Giulia Maga, MSc, RM,  (registered in COMET database 1723) | Care interventions delivered during pregnancy and childbirth  COS for clinical trials or clinical research  COS for practice | - Clinical experts - Consumers (patients) - Methodologists - Policy makers - Researchers - Service users - Statisticians | - Systematic review - Delphi process - Survey - Consensus meeting | Not enough information |
| (36)  PI: Rohan D'Souza  (registered in COMET database 1719) | Pregnancy related studies  COS for clinical trials or clinical research  COS for practice | - Clinical experts - Consumers (caregivers) - Consumers (patients) - Epidemiologists - Families - Journal editors - Methodologists - Patient/ support group representatives - Policy makers - Researchers | - Literature review - Interview - Delphi process - Consensus meeting | Not enough information |
| (37)  PI: Dr Jane Currie  (registered in COMET database 1683) | Urinary tract infection in pregnancy  COS for clinical trials or clinical research  COS for practice | - Charities - Clinical experts - Consumers (patients) - Patient/ support group representatives - Researchers | - Systematic review - Delphi process - Consensus meeting | Not enough information |
| (38)  PI: Clare Whitehead  (registered in COMET database 1670) | COVID-19 and emerging pathogens in pregnancy  COS for clinical trials or clinical research COS for practice Recommendations for outcome measures (measurement/how) | - Clinical experts - Consumers (patients) - Epidemiologists - Families - Patient/ support group representatives - Pharmaceutical industry representatives - Regulatory agency representatives - Researchers - Service providers - Service users | - Systematic review - Focus groups - Survey - Delphi process - Semi structured discussion - Consensus meeting | Not enough information |
| (39)  PI: Rachel Hughes  (registered in COMET database 1648) | Planned mode of birth  COS for clinical trials or clinical research COS for practice | - Clinical experts - Consumers (patients) - Researchers | - Delphi process | Not enough information |
| (40)  Julia Savchenko  (registered in COMET database 1593) | Labour and delivery management at or near term  COS for clinical trials or clinical research COS for practice | - Clinical experts - Patient/ support group representatives - Policy makers - Researchers | - Systematic review - Interview - Delphi process - Consensus meeting |  |
| (41)  Kgosidialwa et al 2020  (registered in COMET database 1425) | Pregnant women with pregestational diabetes mellitus (PGDM)  COS for clinical trials or clinical research | - Patients - Clinicians - Researchers - Policy makers | - Systematic review - Delphi process - Consensus meeting | Yes |
| (42)  PI: Sheryl Green, PhD,  (registered in COMET database 1823) | Anxiety disorders (AD) experienced during pregnancy or the postpartum period  COS for clinical trials or clinical research  COS for practice | - Clinical experts - Conference participants - Consumers (caregivers) - Consumers (patients) - Epidemiologists - Ethicists - Families - Governmental agencies - Methodologists - Patient/ support group representatives - Pharmaceutical industry representatives - Regulatory agency representatives - Researchers - Service providers - Service users - Statisticians | - Systematic review - Focus groups - Interview - Survey - Delphi process - Semi structured discussion - Consensus meeting | Not enough information |

PI: Principal investigator

**Referenser**

1. COMET Database.The CO-OPT Study: Core Outcomes in Obstetric Anal Sphincter Injury Prevention and Treatment. [cited 2020 Jan 31]. Available from: <http://www.comet-initiative.org/studies/details/959> [Internet].

2. COMET Initiative. COMET Database. Network for Preconception and early Pregnancy Randomised Controlled Trialst. [cited 2020 Jan 31]. Available from: <http://www.comet-initiative.org/studies/details/784>.

3. D'Souza R, Hall C, Sermer M, Siu S, Silversides C. Development of a Core Outcome Set for Studies on Cardiac Disease in Pregnancy (COSCarP): a study protocol. Trials. 2020;21(1):300.

4. COMET Initiative. COMET Database. Constructing a core outcome set for Iron Deficiency and Iron Deficiency Anaemia in Pregnancy and Postpartum. [cited 2020 Jan 31]. Available from: <http://www.comet-initiative.org/studies/details/836>.

5. King A, D'Souza R, Teshler L, Shehata N, Malinowski AK. Development of a core outcome set for studies on prevention and management of pregnancy-associated venous thromboembolism (COSPVenTE): a study protocol. BMJ open. 2020;10(7):e034017.

6. COMET Initiative. COMET Database. Constructing a core outcome set for Immune Thrombocytopenia in Pregnancy. [cited 2020 Jan 31]. Available from: <http://www.comet-initiative.org/studies/details/840>.

7. COMET Initiative. COMET Database. Developing a core outcome set for research in multiple pregnancies. [cited 2020 Jan 31]. Available from: <http://www.comet-initiative.org/studies/details/844>.

8. COMET Initiative. COMET Database. Core outcome set for pregnancy outcomes in pregnancies complicated by cancer (COSPOP). [cited 2020 Jan 31]. Available from: <http://www.comet-initiative.org/studies/details/1098>.

9. D'Souza R, Villani L, Hall C, Seyoum M, Kingdom J, Krznaric M, et al. Core outcome set for studies on pregnant women with vasa previa (COVasP): a study protocol. BMJ open. 2020;10(7):e034018.

10. Initiative C. COMET Database. Developing a core outcome set for studies on women with invasive placentation. [cited 2020 Jan 31]. Available from: <http://www.comet-initiative.org/studies/details/1127>.

11. Altoukhi S, Whitehead CL, Ryan G, Deprest J, Joyeux L, Gallagher K, et al. Development of a Core outcome set for fetal Myelomeningocele (COSMiC): study protocol. Trials. 2020;21(1):732.

12. COMET Initiative. COMET Database. Core Outcome Set for Sickle Cell Disease in Pregnancy. [cited 2020 Jan 31]. Available from: <http://www.comet-initiative.org/studies/details/1190>.

13. Killeen SL, O'Brien EC, Jacob CM, O'Reilly SL, Hanson M, McAuliffe FM. PREgnancy Nutrition: A protocol for the development of a Core Outcome Set (PRENCOS). International journal of gynaecology and obstetrics: the official organ of the International Federation of Gynaecology and Obstetrics. 2019;147(2):134-9.

14. Vergote S, De Bie F, Bosteels J, Hedrick H, Duffy J, Power B, et al. Study protocol: a core outcome set for perinatal interventions for congenital diaphragmatic hernia. Trials. 2021;22(1):1-7.

15. Doumouchtsis SK, Rada MP, Pergialiotis V, Falconi G, Haddad JM, Betschart C. A protocol for developing, disseminating, and implementing a core outcome set (COS) for childbirth pelvic floor trauma research. BMC pregnancy and childbirth. 2020;20(1):376.

16. COMET Initiative. COMET Database. Core Outcome Set for Rectovaginal Fistula (RVF). [cited 2020 Jan 31]. Available from: <http://www.comet-initiative.org/studies/details/1082>.

17. COMET Initiative. COMET Database. Development of a Core Outcome Set and identification of outcome measurement tools for interventions for prevention of stillbirth and care after stillbirth (International Collaboration for Harmonising Outcomes fOr Stillbirth resEarch: ICHOOSE). [cited 2020 Jan 31]. Available from: <http://www.comet-initiative.org/studies/details/775>.

18. COMET Initiative. COMET Database. Developing a core outcome set (COS) for clinical trials on interventions for the detection and management of reduced fetal movements in pregnancy. [cited 2020 Jan 31]. Available from: <http://www.comet-initiative.org/studies/details/928>.

19. COMET Initiative. COMET Database. Development of a core outcome set for operative vaginal birth. [cited 2020 Feb 3]. Available from: <http://www.comet-initiative.org/studies/details/1069>.

20. COMET Initiative. COMET Database. The development of a core outcome set for breastfeeding research. [cited 2020 Feb 3]. Available from: <http://www.comet-initiative.org/studies/details/1028>.

21. COMET Initiative. COMET Database. The Composite Adverse Obstetric Outcomes Study (CAOOS). [cited 2020 Feb 3]. Available from: <http://www.comet-initiative.org/studies/details/1154>.

22. Dadouch R, Faheim M, Juando-Prats C, Parsons J, D'Souza R. Development of a Core Outcome Set for Studies on Obesity in Pregnant Patients (COSSOPP): a study protocol. Trials. 2018;19(1):655-.

23. Smith V, Daly D, Lundgren I, Eri T, Begley C, Gross MM, et al. Protocol for the development of a salutogenic intrapartum core outcome set (SIPCOS). BMC Medical Research Methodology. 2017;17(1):61-.

24. Smith P, Cooper N, Dhillon-Smith R, O'Toole E, Clark TJ, Coomarasamy A. Core Outcome Sets in Miscarriage Trials (COSMisT) study: a study protocol. BMJ Open. 2017;7(11):e018535-e.

25. Whitehouse KC, Kim CR, Ganatra B, Duffy JMN, Blum J, Brahmi D, et al. Standardizing abortion research outcomes (STAR): a protocol for developing, disseminating and implementing a core outcome set for medical and surgical abortion. Contraception. 2017;95(5):437-41.

26. Viau-Lapointe J, D’Souza R, Rose L, Lapinsky SE. Development of a Core Outcome Set for research on critically ill obstetric patients: A study protocol. Obstetric Medicine (1753-495X). 2018;11(3):132-6.

27. Prins JR, Holvast F, van 't Hooft J, Bos AF, Ganzevoort JW, Scherjon SA, et al. Development of a core outcome set for immunomodulation in pregnancy (COSIMPREG): a protocol for a systematic review and Delphi study. BMJ Open. 2018;8(8):e021619.

28. Viau-Lapointe J, Lapinsky S, D'Souza R, Kfouri J, Rose L. Development of a core outcome set for studies of pregnant women requiring mechanical ventilation. Journal of Evidence-Based Medicine. 2017;10:24.

29. Sankaran S, Ching-Soh M, Nelson-Piercy C. The urgent need for a multi-disciplinary core outcome set for the reporting of obstetric antiphospholipid antibody syndrome. Trials. 2015;16.

30. COMET Initiative. COMET Database. A protocol for developing and implementing a core outcome set in ectopic pregnancy. [cited 2020 Feb 3]. Available from: <http://www.comet-initiative.org/Studies/Details/1492>.

31. COMET Initiative. COMET Database. Core Outcome Sets for Mental health following Early Pregnancy loss (COSMEP). [cited 2021 Jun 10]. Available from: <https://www.comet-initiative.org/Studies/Details/1763>.

32. COMET Initiative. COMET Database. Development of a core outcome set for effectiveness studies of breech birth at term (Breech-COS): an international multi-stakeholder Delphi study. [cited 2021 Jun 10]. Available from: <https://www.comet-initiative.org/Studies/Details/1749>.

33. COMET Initiative. COMET Database. CRADLE Delphi study: expert Consensus Regarding core outcomes for enhAnced recovery after cesarean DeLivery studiE. [cited 2021 Jun 10]. Available from: <https://www.comet-initiative.org/Studies/Details/1728>.

34. COMET Initiative. COMET Database. Core outcome set for studies of pregnancy affected by multimorbidity. [cited 2021 Jun 10]. Available from: <https://www.comet-initiative.org/Studies/Details/1724>.

35. COMET Initiative. COMET Database. Development and validation of a Midwife-led core outcome set (M-COS) for healthy women and babies. [cited 2021 Jun 10]. Available from: <https://www.comet-initiative.org/Studies/Details/1723>.

36. COMET Initiative. COMET Database. Essential outcomes to be reported in all obstetric studies. [cited 2021 Jun 10]. Available from: <https://www.comet-initiative.org/Studies/Details/1719>.

37. COMET Initiative. COMET Database. Development of a core outcome set for diagnostic and therapeutic studies of urinary tract infection in pregnancy. [cited 2021 Jun 10]. Available from: <https://www.comet-initiative.org/Studies/Details/1683>.

38. COMET Initiative. COMET Database. A core outcome set for COVID-19 and emerging pathogens in pregnancy. [cited 2021 Jun 10]. Available from: <https://www.comet-initiative.org/Studies/Details/1670>.

39. COMET Initiative. COMET Database. Developing a core outcome set for planned mode of birth: a stakeholder consensus process. [cited 2021 Jun 10]. Available from: <https://www.comet-initiative.org/Studies/Details/1648>.

40. COMET Initiative. COMET Database. Swedish perinatal core outcome set (SPeCOS). [cited 2021 Jun 10]. Available from: <https://www.comet-initiative.org/Studies/Details/1593>.

41. Kgosidialwa O, Bogdanet D, Egan A, O'Shea PM, Biesty L, Devane D, et al. Developing a core outcome set for the treatment of pregnant women with pregestational diabetes-a study protocol. Trials. 2020;21(1):1017.

42. COMET Initiative. COMET Database. Developing a Core Outcome Set for Perinatal Women with Generalized Anxiety Disorder (GAD): Standardizing Reported Outcomes in Clinical Research. [cited 2021 Jun 10]. Available from: <https://www.comet-initiative.org/Studies/Details/1823>.
