# Supplementary material for: Core Outcome Sets (COS) related to pregnancy and childbirth: a systematic review
Source: BMC Pregnancy Childbirth. 2021 Oct 9;21:691. doi: 10.1186/s12884-021-04164-y (PMC8501579; doi:10.1186/s12884-021-04164-y)
Supplement: Supplementary file 6 — Additional file 6: Table S4. Compliance with COS-STAR Items. [file 12884_2021_4164_MOESM6_ESM.docx]

Table S4. Compliance with COS-STAR Items

| Number | Question | Nijagal (8) | Bunch (7) | Egan, 2017 (9) | van ʼt Hooft (12) | Devane (17) | Al Wattar (10) | Dos Santos (23) | Briscoe (22) | Fong (13) | Bennett (15) | Rogozinska (11) | Saldanha (14) | Fiala (19) | Bogdanet (6) | Healy (25) | Meher (24) | Perry (26) | Townsend (27) | Mehra (16) | Bashir (1) | Duffy (3) | Egan, 2020 (4) | Gachon (21) | Hellberg (20) | Jansen (5) | Kim (18) | Meissner (2) |
| --- | --- | --- | --- | --- | --- | --- | --- | --- | --- | --- | --- | --- | --- | --- | --- | --- | --- | --- | --- | --- | --- | --- | --- | --- | --- | --- | --- | --- |
| **1a** | Did the title contain information that the paper reports the development of a COS? | Y | P | Y | Y | Y | Y | Y | Y | P | N | P | N | P | Y | Y | Y | Y | Y | P | Y | Y | Y | Y | Y | Y | Y | Y |
| **1b** | Did the abstract contain the key information, such as a list of the recommended outcomes in the COS? | P | Y | P | Y | P | Y | Y | Y | Y | P | Y | N | Y | Y | P | Y | Y | Y | P | P | Y | Y | P | Y | Y | P | P |
| **2a** | Was a background and explanation of the rationale for developing the COS provided? | Y | P | Y | Y | Y | Y | Y | Y | Y | P | Y | P | Y | Y | Y | Y | Y | Y | NA | Y | Y | Y | Y | Y | Y | Y | Y |
| **2b** | Are a clear objective presented? | Y | Y | Y | Y | Y | Y | Y | Y | Y | Y | Y | Y | Y | Y | Y | Y | Y | Y | NA | Y | Y | Y | Y | Y | Y | Y | Y |
| **3a** | Is the *health condition(s)* and *population(s)* covered by the COS sufficiently described? | P | P | Y | Y | P | Y | Y | Y | Y | Y | Y | Y | Y | Y | Y | Y | Y | Y | NA | Y | Y | Y | Y | Y | Y | Y | Y |
| **3b** | Is the *intervention(s)* covered by the COS sufficiently described? | P | P | P | Y | P | P | Y | Y | P | Y | P | Y | Y | Y | Y | Y | Yj | Y | NA | Y | Y | Y | Y | Y | Y | N | Y |
| **3c** | Is the *setting(s)* in which the COS is to be applied sufficiently described? | Y | P | Y | Y | P | Y | Y | Y | Y | Y | P | P | Y | Y | Y | Y | Y | Y | NA | Y | Y | Y | Y | Y | Y | Y | Y |
| **4** | Do the authors provide information about were the COS development protocol can be accessed | N | N | Y | Y | N | Y | Y | N | N | N | N | N | N | Y | Y | N | Y | Y | NA | N | Y | Y | N | Y | N | N | N |
| **5** | Are rationale for which stakeholder groups that are involved and eligibility criteria for participants described? | Y | Y | Y | Y | P | Y | Y | Y | P | P | Y | NA | P | Y | Y | Y | Y | Y | NA | P | Y | Y | P | P | Y | P | P |
| **6a** | Are the information sources used to identify an initial list of outcomes provided? | Y | P | Y | Y | N | Y | Y | Y | Y | N | P | N | Y | Y | Y | Y | Y | Y | NA | Y | Y | Y | P | Y | Y | Y | Y |
| **6b** | Is it described, with reasons, how outcomes were dropped/combined if this was done? | N | N | P | Y | N | Y | Y | P | NA | N | P | N | N | Y | Y | Y | N | Y | NA | P | P | P | P | Y | Y | N | P |
| **7** | Is a description for how consensus process was undertaken provided? | Y | Y | Y | Y | Y | Y | Y | P | Y | P | Y | P | P | Y | Y | Y | Y | Y | NA | Y | Y | Y | Y | Y | Y | Y | Y |
| **8** | Is a description for how outcomes were scored, and scores summarised provided? | P | Y | Y | Y | Y | Y | Y | P | Y | P | Y | P | N | Y | Y | Y | Y | Y | NA | N | Y | Y | P | Y | Y | P | Y |
| **9a** | Is a description of consensus definition provided? | Y | Y | Y | Y | Y | Y | Y | P | P | P | Y | P | N | Y | Y | Y | Y | Y | NA | Y | Y | Y | P | Y | Y | Y | Y |
| **9b** | Is a description for procedure for determining how outcomes were included or excluded from consideration during the consensus process provided | Y | P | Y | Y | Y | Y | Y | P | Y | NA | Y | P | NA | Y | Y | Y | Y | P | NA | P | Y | Y | P | Y | Y | P | Y |
| **10** | Is a statement regarding the ethics and consent issues for the study provided? | Y | Y | Y | Y | Y | N | Y | Y | N | NA | Y | N | N | Y | Y | Y | Y | Y | NA | N | Y | Y | Y | Y | Y | Y | P |
| **11** | Are any changes from the protocol, with reasons, and the impact that these changes may have on the results described? | NA | NA | NA | NA | NA | NA | NA | NA | NA | NA | NA | NA | NA | NA | NA | NA | NA | NA | NA | NA | NA | NA | NA | Y | NA | NA | NA |
| **12** | Are numbers and relevant characteristics of the people involved at all stages of COS development presented? | P | P | Y | Y | Y | Y | Y | Y | Y | Y | Y | P | P | Y | Y | Y | Y | Y | NA | N | Y | Y | Y | Y | Y | Y | Y |
| **13a** | Are all outcomes considered at the start of the consensus process listed? | Y | Y | Y | Y | N | P | Y | Y | Y | N | Y | N | P | Y | Y | Y | Y | Y | NA | Y | N | Y | Y | Y | Y | P | Y |
| **13b** | Are any new outcomes introduced or any outcomes dropped during the consensus process and the reasons for doing so described? | NA | P | Y | Y | P | N | Y | P | P | NA | Y | NA | N | Y | Y | Y | P | Y | NA | P | Y | Y | Y | Y | Y | P | Y |
| **14** | Is a List of the outcomes in the final core outcome set provided? | Y | Y | Y | Y | Y | Y | Y | Y | Y | Y | Y | Y | Y | Y | Y | Y | Y | Y | NA | Y | Y | Y | Y | Y | Y | Y | Y |
| **15** | Are limitations with the COS development process discussed? | Y | Y | Y | Y | Y | Y | Y | Y | Y | Y | Y | P | N | Y | Y | Y | Y | Y | NA | P | Y | Y | Y | Y | Y | Y | Y |
| **16** | Is an interpretation of the final COS in the context of other evidence, and implications for future research given? | P | P | Y | Y | Y | Y | Y | P | Y | P | Y | P | P | Y | Y | Y | Y | Y | NA | Y | Y | Y | Y | Y | Y | Y | P |
| **17** | Is sources of funding and role of funders given? | Y | Y | Y | NA | N | Y | Y | Y | N | Y | Y | Y | Y | Y | Y | Y | Y | Y | NA | Y | Y | Y | Y | Y | Y | Y | Y |
| **18** | Are any conflicts of interest within the study team and how these were managed presented? | Y | Y | Y | Y | N | Y | Y | Y | Y | Y | Y | Y | Y | Y | Y | Y | Y | Y | NA | Y | Y | Y | Y | Y | Y | Y | Y |
| **19** | are all three groups included (researchers, profession and patients) | P | P | Y | Y | Y | P | Y | N | N | N | N | NA | N | Y | Y | Y | Y | Y | NA | N | Y | Y | NA | Y | Y | Y | P |

N= No, P= Partly, Y= Yes, NI= No information available

References

1. Bashir M, Syed A, Furuya-Kanamori L, Musa OAH, Mohamed AM, Skarulis M, et al. Core outcomes in gestational diabetes for treatment trials: The Gestational Metabolic Group treatment set. Obesity Science & Practice. 2021;7(3):251-9.

2. Meissner Y, Fischer-Betz R, Andreoli L, Costedoat-Chalumeau N, De Cock D, Dolhain R, et al. EULAR recommendations for a core data set for pregnancy registries in rheumatology. Ann Rheum Dis. 2021;80(1):49-56.

3. Duffy JMN, Cairns AE, Richards‐Doran D, t Hooft J, Gale C, Brown M, et al. A core outcome set for pre-eclampsia research: an international consensus development study. BJOG: An International Journal of Obstetrics & Gynaecology. 2020;127(12):1516-26.

4. Egan AM, Bogdanet D, Griffin TP, Kgosidialwa O, Cervar-Zivkovic M, Dempsey E, et al. A core outcome set for studies of gestational diabetes mellitus prevention and treatment. Diabetologia. 2020;63(6):1120-7.

5. Jansen L, Koot MH, Van't Hooft J, Dean CR, Duffy J, Ganzevoort W, et al. A core outcome set for hyperemesis gravidarum research: an international consensus study. BJOG : an international journal of obstetrics and gynaecology. 2020;127(8):983-92.

6. Bogdanet D, Reddin C, Macken E, Griffin TP, Fhelelboom N, Biesty L, et al. Follow-up at 1 year and beyond of women with gestational diabetes treated with insulin and/or oral glucose-lowering agents: a core outcome set using a Delphi survey. Diabetologia. 2019.

7. Bunch KJ, Allin B, Jolly M, Hardie T, Knight M. Developing a set of consensus indicators to support maternity service quality improvement: using Core Outcome Set methodology including a Delphi process. BJOG: An International Journal Of Obstetrics And Gynaecology. 2018;125(12):1612-8.

8. Nijagal MA, Wissig S, Stowell C, Olson E, Amer-Wahlin I, Bonsel G, et al. Standardized outcome measures for pregnancy and childbirth, an ICHOM proposal. BMC Health Services Research. 2018;18(1):953-.

9. Egan AM, Galjaard S, Maresh MJA, Loeken MR, Napoli A, Anastasiou E, et al. A core outcome set for studies evaluating the effectiveness of prepregnancy care for women with pregestational diabetes. Diabetologia. 2017;60(7):1190-6.

10. Al Wattar BH, Tamilselvan K, Khan R, Kelso A, Sinha A, Pirie AM, et al. Development of a core outcome set for epilepsy in pregnancy (E-CORE): a national multi-stakeholder modified Delphi consensus study. BJOG : an international journal of obstetrics and gynaecology. 2017;124(4):661-7.

11. Rogozinska E, D'Amico MI, Khan KS, Cecatti JG, Teede H, Yeo S, et al. Development of composite outcomes for individual patient data (IPD) meta-analysis on the effects of diet and lifestyle in pregnancy: a Delphi survey. BJOG: An International Journal of Obstetrics & Gynaecology. 2016;123(2):190-8.

12. van ʼt Hooft J, Duffy JMN, Daly M, Williamson PR, Meher S, Thom E, et al. A Core Outcome Set for Evaluation of Interventions to Prevent Preterm Birth. Obstetrics And Gynecology. 2016;127(1):49-58.

13. Fong F, Rogozinska E, Allotey J, Kempley S, Shah DK, Thangaratinam S. Development of maternal and neonatal composite outcomes for trials evaluating management of late-onset pre-eclampsia. Hypertens Pregnancy. 2014;33(2):115-31.

14. Saldanha IJ, Wilson LM, Bennett WL, Nicholson WK, Robinson KA. Development and pilot test of a process to identify research needs from a systematic review. Journal of Clinical Epidemiology. 2013;66(5):538-45.

15. Bennett WL, Robinson KA, Saldanha IJ, Wilson LM, Nicholson WK. High priority research needs for gestational diabetes mellitus. Journal of women's health (2002). 2012;21(9):925-32.

16. Mehra H, Thangaratinam S. Prioritisation of outcomes in the evaluation of weight management interventions in pregnancy: A DELPHI survey. Archives of Disease in Childhood: Fetal and Neonatal Edition. 2012;97:A38.

17. Devane D, Begley CM, Clarke M, Horey D, Oboyle C. Evaluating maternity care: a core set of outcome measures. Birth (Berkeley, Calif). 2007;34(2):164-72.

18. Kim BV, Aromataris EC, Middleton P, Townsend R, Thangaratinam S, Duffy JMN, et al. Development of a core outcome set for interventions to prevent stillbirth. Aust N Z J Obstet Gynaecol. 2021.

19. Fiala C, Cameron S, Bombas T, Parachini M, Agostini A, Lertxundi R, et al. Outcome of first trimester medical termination of pregnancy: definitions and management. European Journal of Contraception & Reproductive Health Care. 2018;23(6):451-7.

20. Hellberg C, Osterberg M, Jonsson AK, Fundell S, Tronnberg F, Jonsson M, et al. Important research outcomes for treatment studies of perinatal depression: systematic overview and development of a core outcome set. BJOG : an international journal of obstetrics and gynaecology. 2021.

21. Gachon B, Schmitz T, Artzner F, Parant O, De Tayrac R, Ducarme G, et al. A core outcome set development for a French national prospective study about the effect of mediolateral episiotomy on obstetric anal sphincter injury during operative vaginal delivery (INSTRUMODA). BMC pregnancy and childbirth. 2021;21(1):251.

22. Briscoe KE, Haas DM. Developing a Core Outcome Set for Cesarean Delivery Maternal Infectious Morbidity Outcomes. American journal of perinatology. 2019.

23. Dos Santos F, Drymiotou S, Antequera Martin A, Mol BW, Gale C, Devane D, et al. Development of a core outcome set for trials on induction of labour: an international multistakeholder Delphi study. BJOG : an international journal of obstetrics and gynaecology. 2018;125(13):1673-80.

24. Meher S, Cuthbert A, Kirkham JJ, Williamson P, Abalos E, Aflaifel N, et al. Core outcome sets for prevention and treatment of postpartum haemorrhage: an international Delphi consensus study. BJOG: An International Journal Of Obstetrics And Gynaecology. 2019;126(1):83-93.

25. Healy P, Gordijn SJ, Ganzevoort W, Beune IM, Baschat A, Khalil A, et al. A Core Outcome Set for the prevention and treatment of fetal GROwth restriction: deVeloping Endpoints: the COSGROVE study. Am J Obstet Gynecol. 2019;221(4):339.e1-.e10.

26. Perry H, Duffy JMN, Reed K, Baschat A, Deprest J, Hecher K, et al. Core outcome set for research studies evaluating treatments for twin-twin transfusion syndrome. Ultrasound Obstet Gynecol. 2019;54(2):255-61.

27. Townsend R, Duffy JMN, Sileo F, Perry H, Ganzevoort W, Reed K, et al. A core outcome set for studies investigating the management of selective fetal growth restriction in twins. Ultrasound Obstet Gynecol. 2019.
